# Die hard: cell death mechanisms and their implications in nanotoxicology

**DOI:** 10.1093/toxsci/kfad008

**Published:** 2023-02-08

**Authors:** Thanpisit Lomphithak, Bengt Fadeel

**Affiliations:** Division of Molecular Toxicology, Institute of Environmental Medicine, Karolinska Institutet, 171 77 Stockholm, Sweden; Department of Clinical Chemistry, Faculty of Allied Health Sciences, Chulalongkorn University, Bangkok, Thailand; Division of Molecular Toxicology, Institute of Environmental Medicine, Karolinska Institutet, 171 77 Stockholm, Sweden

**Keywords:** apoptosis, ferroptosis, nanomaterials, necroptosis, pyroptosis, toxicology

## Abstract

Cell death is a fundamental biological process, and its fine-tuned regulation is required for life. However, the complexity of regulated cell death is often reduced to a matter of live-dead discrimination. Here, we provide a perspective on programmed or regulated cell death, focusing on apoptosis, pyroptosis, necroptosis, and ferroptosis (the latter three cell death modalities are examples of regulated necrosis). We also touch on other, recently described manifestations of (pathological) cell death including cuproptosis. Furthermore, we address how engineered nanomaterials impact on regulated cell death. We posit that an improved understanding of nanomaterial-induced perturbations of cell death may allow for a better prediction of the consequences of human exposure to these materials and could also yield novel approaches by which to mitigate their effects. Finally, we provide examples of the harnessing of nanomaterials to achieve cancer cell killing through the induction of regulated cell death.

## Introduction

Engineered nanomaterials may interact with biological systems in novel and useful ways. However, they may also perturb these systems leading to adverse outcomes (Fadeel, 2019a). We argued previously that “the nanosafety research community would benefit greatly from applying a molecular diagnosis of cell death engaged by nanomaterials” (Andón and Fadeel, 2013). Indeed, an improved understanding of nanomaterial-induced modulation of cell death may allow for a better prediction and mitigation of the consequences of human exposure. The “pantheon” of programmed cell death modalities has expanded greatly in recent years. Here, we provide an updated perspective on programmed or regulated cell death, focusing on apoptosis, pyroptosis, necroptosis, and ferroptosis. We also provide examples of studies on engineered nanomaterials and each of these modes of cell death. These studies are relevant for our understanding of the undesirable effects of nanomaterials, eg, in workers inadvertently exposed to nanomaterials, and may also be relevant with respect to the desirable properties of nanomaterials, such as immune cell modulation or the killing of cancer cells. Moreover, a detailed understanding of cell death mechanisms is relevant not only in nanotoxicology, but more generally for our understanding of the mechanisms through which foreign substances affect cells and cause tissue damage (Orrenius *et al.*, 2011).

There are currently more than 500 000 articles in PubMed on “apoptosis” and about 5000 articles on each of the major forms of necrosis (ie, pyroptosis, necroptosis, and ferroptosis) (and more than 15 000 entries on the “inflammasome”). The first half of the present review provides an overview of these cell death modalities as well as a critical analysis of cell death research including the methods used (in nanotoxicology). Here, key references, ie, studies that have identified the molecular events underpinning the different cell death modalities are cited along with relevant review articles. In the second half of the review, we discuss these various modes of regulated cell death as they pertain to nanomaterials, focusing on toxicological aspects, followed by a brief discussion of “the other side of the coin” namely how one might exploit this new-found knowledge for therapeutic gain. More than 10 000 articles are listed in PubMed on “nanomaterials” and “apoptosis,” of which about one-third were published in the last 5 years, but far fewer articles are available on the subject of pyroptosis/necroptosis/ferroptosis and nanomaterials (a few hundred articles) (note that we did not consider papers in which nanoparticles were used as carriers of conventional drugs, which in turn triggered cell death, only studies reporting on the intrinsic cell death modulating properties of the particles). We also included some papers on nanomaterials and cell death not cited in PubMed (a database of biomedical literature); thus, relevant publications can also be found in material science journals. We focused on original articles published in the last 5 years, and we selected those studies that fulfilled basic criteria such as: well-characterized nanomaterials and well-described *in vitro* or *in vivo* models (Jeliazkova *et al.*, 2021), and relevant cell death assays (assays which can be used to support claims of a particular cell death modality). The selection was inspired by recent proposals for reporting standards in biotechnology and nanotoxicology (Drobne, 2021; Faria *et al.*, 2018).

## Suicide or sabotage: different forms of regulated cell death

**Figure 1.**
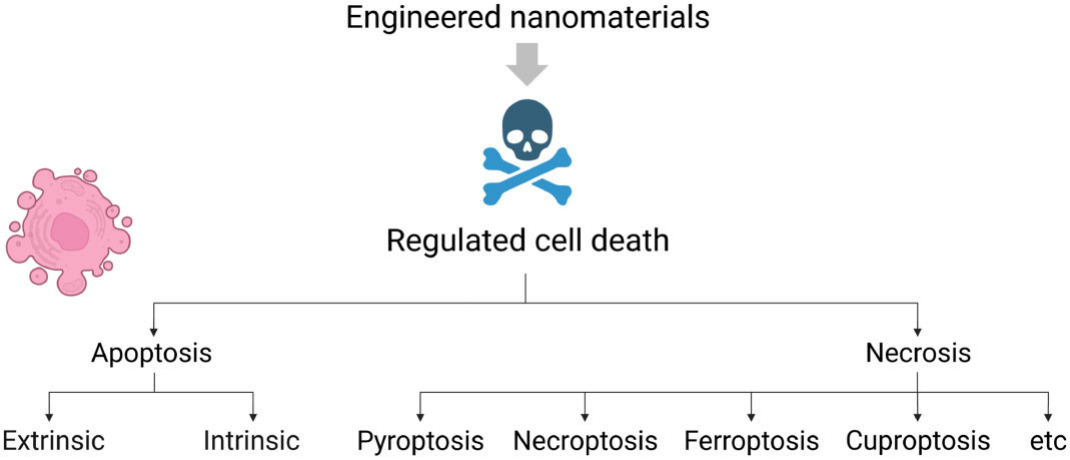
Suicide or sabotage: the pantheon of the fallen. Engineered nanomaterials may trigger different pathways of programmed or regulated cell death. It has been suggested that apoptosis is an active “suicide” program whereas other regulated cell death modalities like ferroptosis constitute a form of cellular sabotage, ie, interference with vital cellular functions such as the protection against oxidative stress (Green and Victor, 2012).

Duvall and Wyllie (1986) concluded, in their prescient essay on cell death, that “cell death is sometimes a pathological event and sometimes a physiological process, apparently as tightly regulated as cell proliferation.” Their discussion was focused mainly on necrosis and apoptosis. However, it appears that this simple dichotomy of apoptosis *versus* necrosis no longer holds water. Moreover, it has been argued that “necrosis” is not a cell death mechanism but refers to the morphological changes that occur secondary to cell death by any mechanism (in other words, necrosis refers to the “features of a cell’s cadaver, whatever the mechanism of the cell’s death”) (Majno and Joris, 1995). Notwithstanding, we now recognize various forms of “regulated necrosis” including necroptosis, pyroptosis, ferroptosis/oxytosis, parthanatos, and others (Vanden Berghe *et al.*, 2014) (Figure 1). To what extent does the (re)discovery of necrosis inform us about the role of cell death in health and disease? It has been argued that the term “regulated” implies that these cell death processes fulfill biological functions, as opposed to being accidental (Fearnhead *et al.*, 2017). However, although apoptosis is clearly a regulated and evolutionarily conserved form of cell death that is required for the development and homeostasis of the organism (Fadeel and Orrenius, 2005), the jury is still out when it comes to other more recently identified forms of regulated cell death such as ferroptosis. Some authors have suggested that ferroptosis is a form of cellular sabotage unlike apoptosis which may be viewed as a *bona fide* cellular “suicide” program (Green and Victor, 2012). The consequences of cell death also matter; in fact, the immunological sequalae of cell death may be more important for the organism than cell death itself (assuming that cell death is balanced by mitosis) (Fadeel, 2019b). The consequences of apoptosis have been studied in detail (Green *et al.*, 2009; Kono and Rock, 2008), but the degree to which ferroptosis triggers immune responses remains poorly understood (Stockwell, 2022). In a recent study, ferroptotic cells were found to dampen antigen presentation by dendritic cells, which seems counterintuitive as ferroptosis is coupled with the release of danger signals that ordinarily would act as adjuvants (Wiernicki *et al.*, 2022). Further studies are warranted to understand the immunological impact of different forms of regulated necrosis.

Programmed cell death is a conserved program of cell elimination and apoptosis is the term used to describe the morphological manifestations of this form of cellular “suicide” (Raff, 1992). Numerous elegant studies using simple model organisms such as nematodes and fruit flies have helped to identify the components of the apoptosis machinery, whereas studies in gene-disrupted mice have served to complement the picture of apoptosis regulation in higher organisms (Danial and Korsmeyer, 2004; Ranger *et al.*, 2001). There is ample evidence that apoptosis plays a role not only in embryogenesis and in tissue homeostasis, but also in a wide spectrum of human diseases. For didactic reasons, apoptosis signaling is divided into 2 main pathways, the “intrinsic” or mitochondria-dependent pathway and the “extrinsic” or death receptor pathway. However, it is clear that many other organelles conspire to trigger apoptosis (Ferri and Kroemer, 2001), leading ultimately to a caspase-dependent dismantling of the cell. One key piece of the apoptosis puzzle was unearthed when the protease that is required for the execution of apoptosis was identified (Nicholson *et al.*, 1995; Tewari *et al.*, 1995). The protease (designated as CPP32, for 32 kDa putative cysteine protease, or apopain/yama), a member of the CED-3/interleukin-lβ-converting enzyme (ICE) family, was found to cleave the “death” substrate poly(ADP-ribose) polymerase (PARP) leading to apoptosis (Nicholson *et al.*, 1995; Tewari *et al.*, 1995). The protease was soon renamed caspase-3 according to the nomenclature for cysteine-dependent, aspartic acid-specific proteases, as the ability to cleave after aspartic acid is the most distinctive catalytic feature of this family of proteases. Another key step in the understanding of the execution of apoptosis was the demonstration that cytochrome c is released from mitochondria to the cytosol soon after induction of cell death, leading to caspase activation, and that the translocation of cytochrome c is blocked by overexpression of BCL-2 (B cell leukemia/lymphoma 2), a member of a large family of pro- and anti-apoptotic proteins (Kluck *et al.*, 1997; Yang *et al.*, 1997). Importantly, a similar blueprint for apoptosis was identified in the nematode, *Caenorhabditis elegans*, thus demonstrating that the “intrinsic” pathway of apoptosis is largely conserved through evolution. The “extrinsic” pathway, on the other hand, seems to be important mainly in the immune system of higher organisms. Here, death signals are transmitted *via* receptors on the cell surface, leading to apoptosis induction. The prototypic death receptor is CD95 (also called Fas or APO-1), and its ligation leads to the activation of the caspase cascade (Krammer, 1998). It is important to note that apoptotic cells are promptly removed by phagocytic cells prior to the rupture of the cell membrane; apoptosis is therefore considered a “silent” cell death.

Pyroptosis is an inflammatory type of regulated cell death driven by caspases 1, 4, 5, and 11. The activation of these caspases in a protein complex called the inflammasome (Martinon *et al.*, 2002) leads to the maturation and release of the pro-inflammatory cytokines interleukin (IL)-1β and IL-18. Hence, pyroptosis was originally defined as “caspase-1-dependent necrosis” and it was also presumed to be a macrophage-specific form of cell death that is prompted by bacterial infections. However, in 2015, cleavage of the protein gasdermin D (GSDMD) by caspase-1 as well as by caspases 4, 5, and 11 was shown to yield a protein fragment that has intrinsic pyroptosis-inducing activity (Kayagi *et al.*, 2015; Shi *et al.*, 2015). The name “gasdermin” was originally based on the expression profile of mouse gasdermin A in the gastrointestinal tract and in the epithelium of the skin (Broz *et al.*, 2020). Caspase-mediated cleavage and activation of GSDMD, but not other gasdermin family members, was thus found to be sufficient to unleash pyroptosis (Kayagi *et al.*, 2015; Shi *et al.*, 2015). In other words, pyroptosis is a form of lytic cell death (necrosis) that is executed by cleavage of GSDMD, a pore-forming protein. However, it is noted that other proteases in addition to caspases can also cleave GSDMD, leading to a cell death that resembles pyroptosis. For instance, neutrophil elastase was shown to cleave GSDMD leading to neutrophil cell death (Kambara *et al.*, 2018). Therefore, it has been suggested that pyroptosis be redefined as “gasdermin-induced necrosis” (Broz *et al.*, 2020). It is notable that caspases are implicated as key executioners in apoptosis and that they also play a role in pro-inflammatory pyroptosis. It is also notable that inflammasomes “sense” a wide range of stimuli including pathogen-associated molecular patterns (PAMPs) as well as endogenous danger signals (also known as DAMPs) such as uric acid and cholesterol crystals, and exogenous substances including crystalline silica, asbestos, and nanomaterials (discussed below).

Necroptosis denotes a form of necrosis that is dependent on receptor-interacting protein kinase 3 (RIPK3) (Degterev *et al.*, 2005, 2008). The first indication of a regulated form of necrosis was demonstrated in the murine L929 fibrosarcoma cell line exposed to tumor necrosis factor (TNF)-α in the presence of a pan-caspase inhibitor (Vercammen *et al.*, 1998). The authors concluded that caspases were involved in “protection” against TNF-α induced necrosis. However, it is not a rescue mechanism if the cells die, and a different interpretation is that necroptosis is the default mode of cell death when caspase-dependent apoptosis is suspended. Notably, although necroptosis is caspase-independent, and is regulated by a distinct set of genes (Hitomi *et al.*, 2008), there is also some interconnectedness between apoptosis and necroptosis, as indicated by the fact that the pro-apoptotic BH3-only protein BMF is also a component of the core machinery for necroptosis (Hitomi *et al.*, 2008). Apart from the TNF receptor, other death receptors and Toll-like receptors transmit necroptosis signals, whereas interferons were found to trigger necrosis through engagement of intracellular protein kinase R (PKR), also known as eukaryotic translation initiation factor 2-alpha kinase 2 (EIF2AK2) (Thapa *et al.*, 2013). PKR has been implicated in inflammasome activation and pyroptosis (Lu *et al.*, 2012), pointing to a degree of cross-talk between different forms of regulated cell death. Furthermore, Z-DNA binding protein 1 (ZBP1) is an intracellular necroptosis-activating sensor of viruses (Zhang *et al.*, 2020b). Thus, necroptosis, like apoptosis, can be activated through death receptors on the cell surface or *via* intracellular receptors. Pyroptosis and necroptosis both transpire with permeabilization of the plasma membrane, and this involves gasdermins in pyroptosis, as discussed above, whereas in necroptosis, lytic cell death requires the oligomerization of mixed lineage kinase domain-like protein (MLKL), a downstream target of RIP3 (Sun *et al.*, 2012; Zhao *et al.*, 2012). In fact, pore formation is a common feature in regulated cell death. Hence, in apoptosis, the permeabilization of the outer mitochondrial membrane by BAX and BAK releases factors like cytochrome c that initiate the caspase cascade (Flores-Romero *et al.*, 2020). In an interesting new twist, Kayagaki *et al.* (2021) have shown that NINJ1, acting downstream of GSDMD, controls plasma membrane rupture in pyroptotic cells, but does not seem to affect secretion of IL-1β and other pyroptosis-associated cytokines. Moreover, *Ninj1^−/−^* mice do not phenocopy *Gsdmd^−/−^* mice in terms of ameliorating LPS-induced sepsis (Kayagaki *et al.*, 2021). It is worth noting that this is an active process, which is separated from cytokine release. In fact, others have shown that membrane repair mechanisms can counterbalance the pores that are formed in regulated necrosis, thereby limiting cytokine secretion (Rühl *et al.*, 2018). Moreover, the cytoprotective amino acid glycine was shown to prevent NINJ1 clustering thus preserving cellular integrity in pyroptotic macrophages (Borges *et al.*, 2022).

Ferroptosis is an iron-dependent, lipid peroxidation-driven form of cell death that is caspase-independent (Dixon *et al.*, 2012). Ferroptosis was identified in a study aimed at understanding the mechanism of action of so-called RAS-selective lethal (RSL) compounds like erastin that selectively kill RAS mutant cells. The authors found that RSL-induced death is associated with increased levels of reactive oxygen species (ROS) and is prevented by iron chelation (Dixon *et al.*, 2012). They also identified a small molecule inhibitor (ferrostatin-1) that prevented ferroptosis in the RAS mutant fibrosarcoma cell line HT-1080. Subsequent work showed that glutathione peroxidase 4 (GPX4) is an essential regulator of ferroptosis (Yang *et al.*, 2014). It is important to note that the inhibition of GPX4 leads to the rapid accumulation of oxidized phospholipids and cell death, implying that (cancer) cells are continually at risk of undergoing lipid peroxidation-mediated destruction. Overall, ferroptosis appears to sit at the “nexus” of cell metabolism (GSH biosynthesis, cellular iron turnover, and lipid metabolism) and cell death (Stockwell *et al.*, 2017). More recent work has shown that ferroptosis suppressor protein 1 (FSP1), previously known as apoptosis-inducing factor mitochondria-associated 2 (AIFM2), confers protection against ferroptosis independent of GSH and GPX4 (Bersuker *et al.*, 2019; Doll *et al.*, 2019).

There is no doubt that ferroptosis has provided necrosis with a new lease on life. The current surge in publications on ferroptosis is akin to the “gold rush” in cell death research several decades ago when the (conserved) genes regulating apoptosis were uncovered (reviewed in: Danial and Korsmeyer, 2004; Meier and Vousden, 2007). However, one may ask whether ferroptosis is a “new” form of cell death or whether we are merely defining the biochemical correlates if not the actual signaling pathways of non-apoptotic cell death aka necrosis? In fact, although the term ferroptosis was coined 10 years ago (Dixon *et al.*, 2012), advances in cell death research that contributed to the concept of ferroptosis have emerged over a period of several decades (Hirschhorn and Stockwell, 2019). Hence, lipid peroxidation and glutathione (GSH) depletion are well known features of necrosis, and the role of iron in lipid peroxidation was described half a century before the term ferroptosis was coined (Hirschhorn and Stockwell, 2019), whereas the eponymous Fenton reaction was described in the 1890s. As pointed out by Hirschhorn and Stockwell (2019), “this reaction partially explains the dependency of ferroptosis on iron, as redox-active iron pools are able to directly catalyze propagation of lipid peroxidation to form damaging species that lead to cell death.” The lysosomes were dubbed the “suicide bags” of the cell by de Duve (Turk and Turk, 2009), and it is important to note that these organelles contain a substantial proportion of the labile iron pool (LIP) consisting of redox-active Fe^2+^ (Rizzollo *et al.*, 2021). Indeed, using unbiased genetic screens, investigators have found that the key role of lysosomes is to maintain iron homeostasis in the cell (Weber *et al.*, 2020). Therefore, it is unsurprising that lysosomes have been found to play a role in ferroptosis (Torii *et al.*, 2016). However, the introduction of the term ferroptosis provided a framework for the understanding of non-apoptotic, lipid peroxidation-driven cell death (Stockwell *et al.*, 2017). Furthermore, GPX4 was shown to play a role in protecting membranes from iron-catalyzed lipid peroxidation already in the 1980s (Hirschhorn and Stockwell, 2019). Another important precursor to ferroptosis was the study by Seiler *et al.* (2008) showing that the deletion of GPX4 in mice caused a non-apoptotic, redox-dependent form of cell death characterized by 12/15-lipoxygenase-mediated lipid peroxidation. Notably, the cell death was apoptosis-inducing factor (AIF)-dependent (Seiler *et al.*, 2008), suggesting that AIF is also a “necrosis-inducing factor” that plays a key role in non-apoptotic cell death. Indeed, AIF and its associated nuclease, macrophage migration inhibitory factor (MIF) (rechristened as PARP-1-dependent AIF-associated nuclease or PAAN), are key players in “parthanatos” (Kam *et al.*, 2018; Wang *et al.*, 2016), a regulated necrosis implicated in neurodegenerative disease.

## Dire straits: pitfalls and misconceptions in cell death research

Yogi Berra once said, “you can observe a lot by watching,” and it is true that cell death was originally defined based on morphological criteria (Majno and Joris, 1995; Trump *et al.*, 1997). The “discoveries concerning genetic regulation of organ development and programmed cell death” for which Sydney Brenner, Robert Horvitz, and John Sulston were awarded the Nobel Prize in Physiology or Medicine broke the mold—cell death had finally achieved the status of a *bona fide* genetically regulated biological process. However, students of cell death would be amiss to ignore the vast amount of information that awaits under the microscope—the net result of all those genes and proteins. Cell death has been widely studied not least in (nano)toxicology yet misinterpretations with respect to commonly used cell viability or cell death assays prevail (Özkaya and Geyik, 2022). In fact, some popular assays are frequently used as a proxy for specific cell death modalities like apoptosis even when this is not justified. For instance, the annexin V-based assay that is used to label cells that display phosphatidylserine (PS) on the cell surface is often used as a specific apoptosis assay, but this may lead to an underestimation of other forms of cell death including necroptosis in which PS is externalized albeit in a caspase-independent manner (Klöditz and Fadeel, 2019). Furthermore, annexin V also binds to cardiolipin, a mitochondrial phospholipid that is sometimes exposed on the cell surface (Balasubramanian *et al.*, 2015). Even the use of vital dyes like trypan blue to monitor the loss of plasma membrane integrity has been called into question (Husmann, 2013) (not so much the assay itself as whether loss of plasma membrane integrity is a useful criterion by which to define cell death, given that cells may repair holes in the plasma membrane). In nanotoxicology, there is another concern namely that nanomaterials may interfere with many cell viability assays (MacCormack *et al.*, 2021; Ong *et al.*, 2014).

It is important to recognize that there is no single assay that can be used to make the diagnosis of regulated cell death. Indeed, whereas apoptosis has been morphologically and biochemically defined to a fault, this is not true for other forms of regulated cell death which are classified as “non-apoptotic” forms of cellular demise. DNA fragmentation is a hallmark of apoptosis and thus represents a convenient marker of this form of cell death (Wyllie, 1980). Moreover, the identification of caspase-3, the key executioner of apoptosis, and its cellular substrates (Whyte and Evan, 1995), has enabled the unequivocal detection of apoptosis in cells and tissues. In contrast, commonly used assays for necrosis, such as the lactate dehydrogenase (LDH) release assay, or vital dye exclusion assays (Monteiro-Riviere *et al.*, 2009), are not specific for any particular form of cell death. Therefore, studies of regulated necrosis must be conducted using pharmacological and/or genetic approaches to pinpoint the mode of cell death. Moreover, the toxicological evaluation of nanomaterials should take into account the kinetics of the response (the initial induction of a cytoprotective autophagic response may be followed by apoptosis, or necrosis, at a later time-point) and the dose (the same stimulus may trigger apoptosis when administered at a low dose and necrosis if administered at a high dose). Indeed, even the apparently simple question of the “dose” (in an *in vitro* system) is not so simple, and it is important to realize the distinction between the nominal dose *versus* the delivered (or received) dose (Lison *et al.*, 2014). For instance, cytotoxicity may be underestimated for buoyant nanoparticles displaying a density lower than that of the cell medium (Watson *et al.*, 2016).

Lysosomes are key organelles in cell death, and they appear to be of particular importance for cell death triggered by nanoparticles (Andón and Fadeel, 2013). This is not surprising given that nanoparticles are often internalized through an endocytic mechanism and trafficked to the lysosomes. In contrast, in our experience, nanoparticles are rarely found in mitochondria. It is important, however, to point out that although cell death studies are frequently performed using cancer cell lines, these cells may suffer from perturbations in the very pathways that are under study; after all, one of the hallmarks of cancer is resisting cell death (Hanahan, 2022). Furthermore, changes in the structure and function of lysosomes are commonly found in cancer cells (Iulianna *et al.*, 2022). Indeed, it has been suggested that “the function of lysosomes is often adaptively or maladaptively upregulated to meet the metabolic requirements of cancer cells” (Du *et al.*, 2021). This has implications for cancer therapy but also for toxicological investigations of nanomaterials (in other words, we need to think twice before extrapolating results from transformed cell lines to normal cells).

Necroptosis can be defined as a non-apoptotic cell death that is blocked by necrostatin-1 (Vanden Berghe *et al.*, 2014). Necrostatin-1 was identified in a small molecule screen for inhibitors of necrotic death of human monocytic U937 cells induced by TNF-α and zVAD-fmk, which was used as an operational definition of “necroptosis” (Degterev *et al.*, 2005). The molecular target was identified as receptor-interacting serine/threonine-protein kinase 1 (RIPK1) kinase (Degterev *et al.*, 2008). Ferroptosis, in turn, was described as a non-apoptotic cell death in a seminal study by Dixon *et al.* (2012), and the small molecule ferrostatin-1 was identified as a potent inhibitor of ferroptosis—evidenced as erastin-induced cell death in the human fibrosarcoma cell line HT-1080. Erastin, also identified in a chemical screen, is a compound that is selectively lethal to oncogenic RAS mutant cell lines (Yang and Stockwell, 2008). Thus, although the molecular target for erastin is well defined, this is not necessarily true for inhibitors like ferrostatin-1 and liproxstatin-1 that are believed to act as radical-trapping antioxidants (Zilka *et al.*, 2017). Hence, the latter inhibitors are not “diagnostic.” Indeed, if we define cell death based on whether it is blocked by certain inhibitors as is often the case in the toxicological literature then we run into a problem if the same molecule or signaling pathway is involved in more than one form of cell death (or in case of cross-talk between different cell death modalities) (Kist and Vucic, 2021). Moreover, some commonly used inhibitors may display off-target effects. For instance, necrostatin-1 also inhibits the immunomodulatory enzyme indoleamine 2,3-dioxygenase (IDO) (Takahashi *et al.*, 2012). Additionally, although ferroptosis implies a non-apoptotic cell death that is iron-dependent by nature, iron may also play a role in other modes of cell death (Soriano-Castell *et al.*, 2021). Moreover, lipid peroxidation is not unique to ferroptosis. On the other hand, it is important to distinguish between events that are critical for the execution of cell death *versus* those that occur as a consequence thereof. Based on a systematic comparison of apoptosis, necroptosis, ferroptosis, and pyroptosis, it has been argued that ferroptosis is the only mode of regulated cell death in which phospholipid peroxidation drives cell death (Wiernicki *et al.*, 2020). It is also entirely possible that the pattern of lipid peroxidation in ferroptosis is specific (Stoyanovsky *et al.*, 2019), although much more work is needed to decipher the lipid “alphabet” in cells and tissues.

Box 1. Pandora’s box: regulated cell death. Cell death is manifested in many ways and novel signaling pathways that drive regulated cell death (RCD) continue to emerge. The Nomenclature Committee on Cell Death (NCCD) has formulated guidelines on the definition and interpretation of RCD (see: Galluzzi *et al.*, 2018, for the most recent iteration). Here, we provide a summary and an update on the most well-studied cell death modalities.

**Apoptosis:** a caspase-dependent cell death that transpires through the extrinsic or intrinsic route; the former is triggered by cell death receptors and propagated by caspase-8 whereas the latter is mitochondria-dependent and propagated by apoptosome-driven caspase-9 activation. Both apoptosis pathways are precipitated by executioner caspases mainly caspase-3. BCL-2 family proteins regulate apoptosis.

**Autophagic cell death:** a form of RCD that depends on the autophagic machinery (to resolve this unfortunate circular argument, refer to: Klionsky *et al.*, 2021). “Entosis,” a form of cellular cannibalism, displays autophagic features (Overholtzer *et al.*, 2007).

**Pyroptosis:** RCD characterized by the formation of plasma membrane pores by members of the gasdermin family following the activation of inflammatory caspases (eg, caspase-1). The cytosolic inflammasome complex plays a key role in pyroptosis.

**Necroptosis:** a modality of RCD triggered through death receptors or intracellular perturbations that depends on the sequential activation of RIPK3 and MLKL and (sometimes) on RIPK1. The RIPK1-RIPK3-MLKL complex is referred to as the necrosome.

Notably, a recent study has suggested a common feature shared by cells dying of pyroptosis, necroptosis, and secondary necrosis following apoptosis, namely NINJ1-dependent rupture of the plasma membrane (Kayagaki *et al.*, 2021). This releases cytoplasmic constituents, including so-called DAMPs (reviewed in: Newton *et al.*, 2021).

**Ferroptosis:** an iron-dependent form of RCD characterized by lipid peroxidation that is under constitutive control by cellular antioxidant systems (eg, GPX4) (Stockwell, 2022). Cysteine deprivation, eg, through system x_c_ inhibition, also triggers ferroptosis. “Oxytosis” is a calcium-dependent form of glutamate-induced neuronal cell death that is initiated by system x_c_ inhibition and GSH depletion and is reminiscent of ferroptosis.

**Cuproptosis:** a non-apoptotic cell death (in cancer cells) triggered by excess copper which is associated with the direct binding of copper to lipoylated components of the tricarboxylic acid (TCA) cycle resulting in protein aggregation, and proteotoxic stress.

**Parthanatos:** RCD defined by PARP-1 hyperactivation and subsequent AIF-dependent DNA degradation. The term is a portmanteau of *PAR*, for poly (ADP-ribose), a product of PARP-1, and “Thanatos,” the personification of death in Greek mythology.

Other manifestations of cell death, not (yet) sanctioned by the NCCD, have been identified, eg, “oxeiptosis” (Holze *et al.*, 2018), “calcicoptosis” (Zhang *et al.*, 2019), and “lysozincrosis” (Du *et al.*, 2021). It seems that Pandora’s box is full of surprises, but it remains to be understood whether these phenomena can be generalized (Green, 2019).

## Fire in the hole: nanomaterials and (de)regulated cell death

We have previously discussed the complexities of nanomaterial-induced perturbations of cell death pathways (Andón and Fadeel, 2013). In the following section, we will provide an updated perspective on various cell death mechanisms triggered by nanomaterials. For didactic reasons, we shall discuss one cell death modality at a time. However, it is worth considering that different cell death pathways do not necessarily operate in isolation (Bedoui *et al.*, 2020). Indeed, there may be a degree of overlap or back-up between cell death programs, and cells may not be as preoccupied with cell death classifications as cell death researchers (Galluzzi *et al.*, 2018). For a summary of current definitions of various cell death modalities, see Box 1.

Numerous studies during the past decade have shown that nanomaterials can trigger apoptosis, as suggested by the involvement of the “killer” caspases. These studies cover a range of different nanomaterials including single-walled carbon nanotubes, metallic nanoparticles such as Ag nanoparticles, and metal oxides such as TiO_2_ and ZnO, perhaps reflecting the fact that these belong to the most studied nanomaterials to date. For instance, several studies have disclosed that Ag nanoparticles provoke endoplasmic reticulum (ER) stress leading to caspase-dependent apoptosis (Ho *et al.*, 2015; Zhang *et al.*, 2012). However, Ag nanoparticles may undergo dissolution, and it is pertinent to ask whether the mechanism of cell death differs for Ag in its particulate form *versus* soluble Ag^+^ ions. Using a triple-negative breast cancer cell line as a model, Rodhe *et al.* (2021) recently found distinct differences in cellular responses to Ag nanoparticles with very limited dissolution and Ag^+^ ions. Specifically, the nanoparticles were found to drive cell death through lipid peroxidation leading to proteotoxicity and necrotic cell death, whereas Ag^+^ ions increased cellular hydrogen peroxide levels leading to oxidative stress and apoptotic cell death. Others have shown that Ag nanoparticles but not the released Ag^+^ ions triggered genotoxicity through oxidative stress in a lymphoblastoid cell line (Li *et al.*, 2017). Gold (Au) nanoparticles were also shown to trigger ER stress followed by mitochondria-dependent apoptosis, in a study using the human chronic myelogenous leukemia K562 cell line (Tsai *et al.*, 2011), whereas other investigators have reported that Au nanoparticles induced the accumulation of autophagosomes in normal rat kidney epithelial cells (Ma *et al.*, 2011). Clearly, the doses applied will play an important role, and the use of normal (primary) cells *versus* transformed cell lines will also impact on the outcome of any nanotoxicity test. Furthermore, surface functionalization of the nanoparticles may have a profound impact on the cellular responses (Gallud *et al.*, 2020). We observed a pronounced cytotoxicity for ammonium-terminated Au nanoparticles using the THP-1 cell line as a model, whereas no cell death was seen after exposure to carboxylated or PEG-modified Au nanoparticles (Gallud *et al.*, 2019). The Au nanoparticles triggered caspase-dependent cell death (apoptosis) in THP-1 cells; moreover, cell death was aggravated upon inhibition of autophagy. However, when the dose of Au nanoparticles was increased, the cytoprotective autophagic response was overwhelmed and the cells succumbed to a necrotic cell death without any signs of caspase activation. This was confirmed *in vivo* using *C. elegans* as a model. Hence, we found that loss of *clp-1* (required for necrosis) but not loss of *ced-3* (required for apoptosis) or *lgg-1* (required for autophagy) reduced lethality induced by a high dose of the ammonium-terminated Au nanoparticles, whereas exposure to the carboxylated Au nanoparticles did not cause any lethality to the larvae (Gallud *et al.*, 2019). Other investigators have shown that the “switch” between necrosis and apoptosis could be realized by tailoring the surface properties of Au nanorods (Zhang *et al.*, 2021). Hence, the authors found that Au nanorods with small surface molecule densities mainly induced apoptosis, whereas those with large surface molecule densities contributed to necrosis. Evidence was provided that the interaction between cetyltrimethylammonium bromide (CTAB)-capped Au nanorods and lysosomes caused the upregulation of caspase-8 which, in turn, cleaved or otherwise blocked RIP1–RIP3, leading to caspase-3 activation and, hence, a “switch” from necrosis to apoptosis (Zhang *et al.*, 2021). The authors also suggested that the release of cathepsin B contributed to necrosis whereas cathepsin D evoked apoptosis, but the appearance of these proteases in the cytosol cannot be taken as evidence for their involvement in apoptosis or necrosis, and it may be difficult to disentangle the role of specific lysosomal proteases in different cell death programs. Indeed, nanoparticles might trigger a “mixed” cell death with features of different forms of regulated cell death.

He *et al.* (2018) investigated a set of carbon-based nanomaterials including single-walled carbon nanohorns (SNH) *versus* single-walled and multi-walled carbon nanotubes (CNTs) with respect to cell death mechanisms. SNH monomers assemble to form globular aggregates; hence, these materials are the spherical equivalents of carbon nanotubes. Using the murine macrophage-like J774A.1 cell line, the authors found less uptake of SNH when compared with CNTs, and SNH were also less cytotoxic. Additionally, the CNTs, but not SNH, triggered caspase-dependent apoptosis, as well as signs of pyroptosis-mediated necrosis with IL-1β release. Finally, it was shown using a proteomics approach that the different nanocarbons interacted with cellular proteins including the transmembrane protein known as glycoprotein nonmetastatic melanoma protein B (GPNMB), whereas silencing of GPNMB protected the cells from CNT-induced cell death (He *et al.*, 2018). Thus, this comprehensive study has shown that specific cellular receptors may trigger nanotoxicity in macrophages and that the geometry of the nanomaterials plays an important role. The study also provided an instructive example of a “mixed” cell death (ie, caspase-3-driven apoptosis plus caspase-1-driven pyroptosis) in response to nanomaterials whereas other forms of cell death including necroptosis could be excluded.

**Figure 2.**
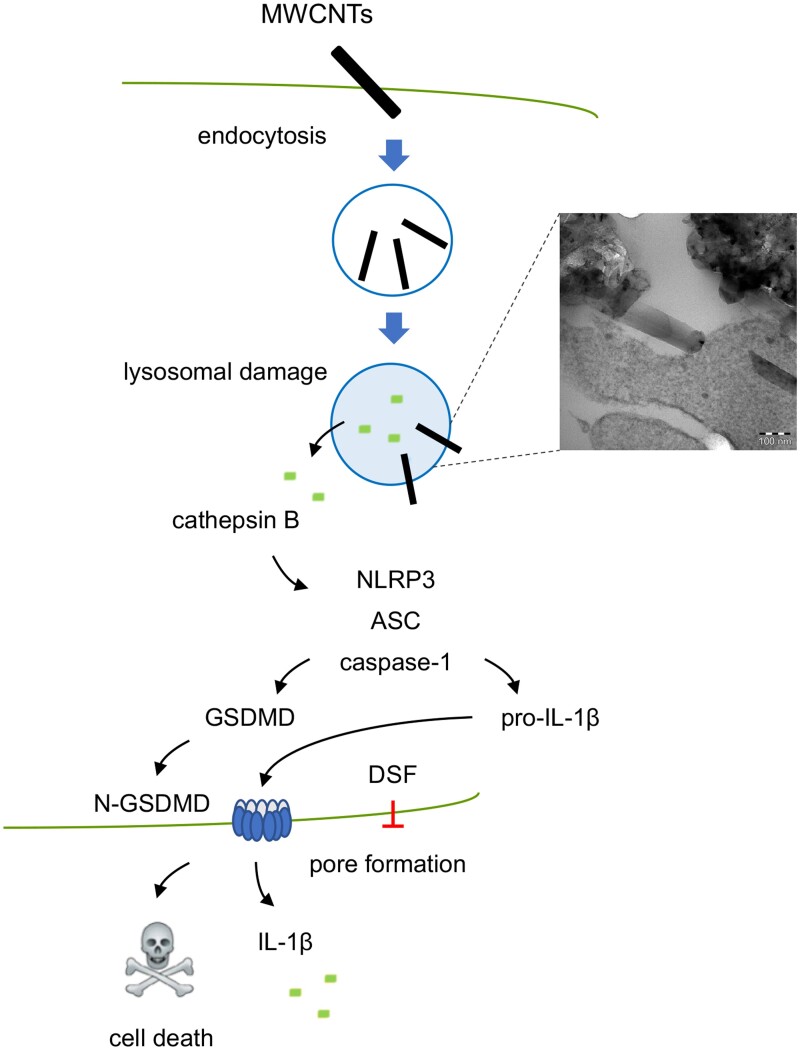
Fire in the hole: long and rigid carbon nanotubes trigger pyroptosis. Using a set of multi-walled carbon nanotubes (MWCNTs) with varying morphology (long and rigid vs short and/or tangled), Keshavan *et al.* (2021) showed that the long and rigid nanotubes triggered cathepsin B-dependent cell death in macrophages, accompanied by NLRP3 inflammasome activation and gasdermin D (GSDMD)-mediated release of pro-inflammatory IL-1β. The IL-1β release was suppressed by disulfiram (DSF), an FDA-approved drug known to act as an inhibitor of pore formation by GSDMD. N-GSDMD, N-terminal fragment of GSDMD, generated by caspase-1 cleavage. Others have shown that rare earth oxides (REO) trigger pyroptosis in macrophages and Kupffer cells through a similar pathway (Mirshafiee *et al.*, 2018). Scale bar: 100 nm. Modified from Keshavan *et al.* (2021) with permission from Taylor & Francis.

Nanotoxicological studies are frequently performed using cancer cell lines. However, we recently investigated the mode of cell death triggered by multi-walled carbon nanotubes using both primary human monocyte-derived macrophages and the macrophage-differentiated THP-1 cell line as well as primary human neutrophils and the neutrophil-differentiated HL-60 cell line (Keshavan *et al.*, 2021). These studies demonstrated that long and rigid nanotubes (NM401) but not the short and/or tangled nanotubes (NM400 and NM402) triggered pyroptosis in macrophages, but not in neutrophils (Figure 2). We provided evidence for a role of lysosomal cathepsin B release, leading to NLRP3-dependent caspase-1 activation with GSDMD cleavage, followed by IL-1β release. We could also show that disulfiram, an “old” drug that was recently shown to inhibit pore formation by GSDMD (Hu *et al.*, 2020), blocked IL-1β release in cells exposed to the long and rigid CNTs. Mirshafiee *et al.* (2018) performed a comparative analysis of the impact of a panel of metal oxide nanoparticles using a murine hepatocyte cell line (Hepa 1–6) *versus* a Kupffer (liver-resident macrophage) cell line (KUP5). The authors found significant differences between transition metal oxides (TMOs, eg, Co_3_O_4_) and rare earth oxides (REOs) NPs (eg, Gd_2_O_3_). Thus, although TMOs induced the activation of caspases 3 and 7, resulting in apoptotic cell death in both cell types, REOs induced lysosomal damage, NLRP3 inflammasome activation, caspase-1 activation, and pyroptotic cell death in the Kupffer cell line as well as in other macrophage cell lines, but not in the hepatocyte cell line. Notably, knockdown of GSDMD ameliorated REO-induced cell death in KUP5 cells, confirming a role for pyroptosis. Moreover, REOs also triggered IL-1β release in primary human Kupffer cells. In a follow-up study, the authors, focused on a panel of metal, metal oxide, and metal sulfide nanoparticles, using the same cell lines. The results showed that Ag, CuO, and ZnO nanoparticles triggered caspase-3-mediated apoptosis in both cell lines, whereas SiO_2_ nanoparticles triggered pyroptosis in the Kupffer cell line, with potassium efflux, NLRP3 inflammasome assembly, caspase-1 activation, GSDMD cleavage, and release of IL-1β. Several other studies have shown that nanomaterials provoke inflammasome activation (Farrera and Fadeel, 2015). For instance, multi-walled carbon nanotubes (MWCNT) were shown to trigger NLRP3 inflammasome activation in macrophages (Palomäki *et al.*, 2011). MWCNT were also shown to trigger NRLP3 inflammasome activation in primary human bronchial epithelial cells and the authors suggested that this might contribute to fibrotic responses (in the lungs) (Hussain *et al.*, 2014). Sun *et al.* (2015) found that NADPH oxidase activation is critically involved in inflammasome activation, as evidenced by the fact that MWCNT-induced NLRP3 inflammasome activation and pulmonary fibrosis were attenuated in p47^phox^-deficient mice compared with wild-type mice. However, inflammasome activation is not always coupled with cell death, as we have shown for hollow carbon spheres and primary human monocyte-derived macrophages (HMDM) (Andón *et al.*, 2017). Similarly, we also found that graphene oxide triggers NLRP3 inflammasome activation in HMDM in the absence of cell death (Mukherjee *et al.*, 2018).

NLRP3 has emerged as a key “sensor” of microbial and nonmicrobial agents, and there is an extensive body of work on the various biochemical cues and signaling events leading to NLRP3 inflammasome activation (Swanson *et al.*, 2019). Interestingly, physical cues may also trigger inflammasome activation. Wang *et al.* (2018) synthesized TiO_2_ microparticles (approximately 1.8 µm) decorated with nanospikes (approximately 20 nm in diameter) and were able to show that the “spiky” particles specifically activated inflammasomes by stimulating cellular potassium efflux, possibly due to a nanospike-mediated mechanical stress on the cell membrane. To generate particles with a rough topography without any nanospikes for comparison, the authors sonicated the “spiky” particles up to 20 h. The “spiky” particles also promoted dendritic cell maturation *in vitro* and *in vivo*. It is notable, as pointed out by the authors, that many pathogens have spike-like protrusions on their surface, but it is challenging to separate the biological from the structural cues on the surface of, for instance, a virus. However, the present study using artificial (non-biological) particles as a model suggests that physical cues may be exploited to improve the adjuvanticity of vaccines (Wang *et al.*, 2018). Li *et al.* (2014a) provided a particularly instructive example of nanoparticle-induced inflammasome activation. Using *in vitro* and *in vivo* models, the authors could show that ion shedding by REOs in lysosomes caused organelle damage due to the stripping of phosphates from the lipid membranes of the cells, which, in turn, triggered NLRP3 inflammasome activation and IL-1β release. In contrast, TiO_2_ nanoparticles did not elicit such effects. However, pretreatment of REOs, exemplified by La_2_O_3_ nanoparticles, with phosphate in a neutral pH environment prevented the biotransformation and pro-fibrogenic effects of these nanoparticles. In a companion study, REO nanoparticles interfered with the fusion of autophagosomes with lysosomes (Li *et al.*, 2014b). This resulted in the accumulation of activated inflammasomes and sustained IL-1β production. Thus, although some nanomaterials promote the induction of autophagy, other nanomaterials such as REOs seem to interfere with autophagic flux, thereby disrupting inflammasome regulation (Li *et al.*, 2014b).

There are comparatively few studies on engineered nanomaterials and necroptosis. Sagawa *et al.* (2021) reported that intratracheally administered TiO_2_ nanoparticles induced acute lung inflammation in mice with evidence of necroptosis of alveolar macrophages. Hence, alveolar macrophages that had engulfed nanoparticles expressed phosphorylated MLKL and displayed translocation of HMGB1, a well-known DAMP, from the nucleus to the cytoplasm. Furthermore, necrostatin-1 was shown to suppress cell death of alveolar macrophages *in vivo* as well as in primary alveolar macrophages cultivated *ex vivo*. However, it is noted that the authors applied very high doses of nanoparticles (ie, 200 µg per mouse, and 200 µg/mL for *ex vivo* studies). Moreover, only rutile but not anatase particles were investigated. Several intrinsic crystalline particles including monosodium urate (MSU) or cholesterol crystals, along with extrinsic variants such as crystalline silica or quartz, have been shown to trigger the NLRP3 inflammasome (see above). However, while inflammasome activation is not always coupled with cell death, evidence has been provided that the cytotoxicity elicited by crystalline particles is RIPK-MKLK-dependent (Mulay *et al.*, 2016). In a subsequent study, a broad range of environmental and metabolic crystalline particles were shown to induce RIPK1-RIPK3-MLKL-mediated necroptosis using human and murine renal tubular cells as a model system (Honarpisheh *et al.*, 2017). Furthermore, using a series of different particles including crystals of MSU, cholesterol, and silicon dioxide (silica), it was shown that crystalline particles triggered necroptosis in human and murine neutrophils along with the release of neutrophil extracellular traps (NETs) (Desai *et al.*, 2017). Mice deficient in MLKL showed drastically reduced NET formation upon exposure to all the particles, confirming the involvement of necroptosis in crystal-induced cell death. These studies suggest that necroptosis could be a potential target in crystallopathies. Interestingly, nanodiamonds were shown to trigger NET release in a size-dependent fashion *in vitro* and *in vivo* (Munoz *et al.*, 2016). However, very high doses were applied; thus, 1 mg of nanoparticles were injected into the foot pad of mice, whereas 200 µg/mL of particles were added to isolated neutrophils. Further studies are warranted to test whether nanoparticle-induced necroptosis is also dependent on shape and/or crystal structure.

The oxidative stress paradigm is frequently invoked to explain the toxic potential of particulate matter including engineered nanoparticles (Nel *et al.*, 2006). However, oxidative stress could potentially lead to distinct cellular outcomes depending on the cell and/or nanoparticle type (as well as on the dose and duration of the stimulus). Indeed, Szwed *et al.* (2019) have shown, in a comprehensive *in vitro* study, that small variations in the structure of nanoparticles may have a profound impact on the mode of cell death. The authors focused their attention on nanoparticles of poly(alkylcyanoacrylate) (PACA), originally used as surgical glue, with differences in their alkyl side chains, ie, butyl (PBCA), ethylbutyl (PEBCA), or octyl (POCA). The nanoparticles all displayed similar mean diameters (130–150 nm) and were slightly negatively charged with a ζ-potential of −3 mV. POCA particles triggered apoptosis with caspase-3-dependent cleavage of PARP. In contrast, PBCA particles induced a non-apoptotic cell death which was reversed by ferrostatin and liproxstatin as well as by the iron chelator, deferiprone, and lipid peroxidation was also documented, suggesting that these particles triggered ferroptosis. In cancer cells subjected to cystine-starvation (cystine is converted to cysteine, which is the rate-limiting substrate in GSH synthesis), both PBCA and PEBCA triggered ferroptosis. The authors noted that the three polymeric particles differed in that their alkyl side chains showed increasing hydrophobicity (PBCA < PEBCA < POCA) which could potentially drive distinct interactions of these nanoparticles with biological structures (Szwed *et al.*, 2019). We found that cobalt nanoparticles and soluble CoCl_2_ triggered a dose-dependent cytotoxicity with a marked depletion of GSH levels in the human neuron-like cell line SH-SY5Y as well in primary human iPSC-derived dopaminergic neurons (Gupta *et al.*, 2020). Lipid peroxidation was documented in SH-SY5Y cells. The caspase inhibitor zVAD-fmk and RIPK inhibitor necrostatin-1 failed to rescue the cells, but deferoxamine, an iron chelating agent, afforded partial protection, suggesting a ferroptosis-like cell death (also termed oxytosis) in cobalt-exposed neurons. Ferroptosis-like cell death has been confirmed using the BALB/3T3 fibroblast cell line, and the antioxidant α-lipoic acid antagonized the effects of the cobalt nanoparticles (Liu *et al.*, 2020). Several studies have been published in recent years in which nanomaterials were found to trigger ferroptosis, the overwhelming majority of which address cancer cell killing (see below). Xu *et al.* (2020) showed that two-dimensional (2D) transition metal dichalcogenides (TMDs), exemplified by WS_2_ and MoS_2_ nanosheets, induced an iron-dependent cell death in human lung-derived (BEAS-2B) and macrophage-like (THP-1) cells. The authors provided evidence for lysosomal Fe^2+^ release into the cytoplasm triggering ROS generation and lipid peroxidation, leading ultimately to cell death. However, it is noted that these effects were observed at high doses (200 µg/mL) of TMDs. In contrast, we have recently shown that nanosheets of WS_2_ and MoS_2_ were noncytotoxic towards primary human monocyte-derived macrophages (Peng *et al.*, 2022). These discrepancies could perhaps be explained by differences in the synthesis and physicochemical properties of the TMDs, and differences between primary cells and cell lines, and differences in the dose.

## Two sides of the coin: nanoparticles as therapeutic agents

**Figure 3.**
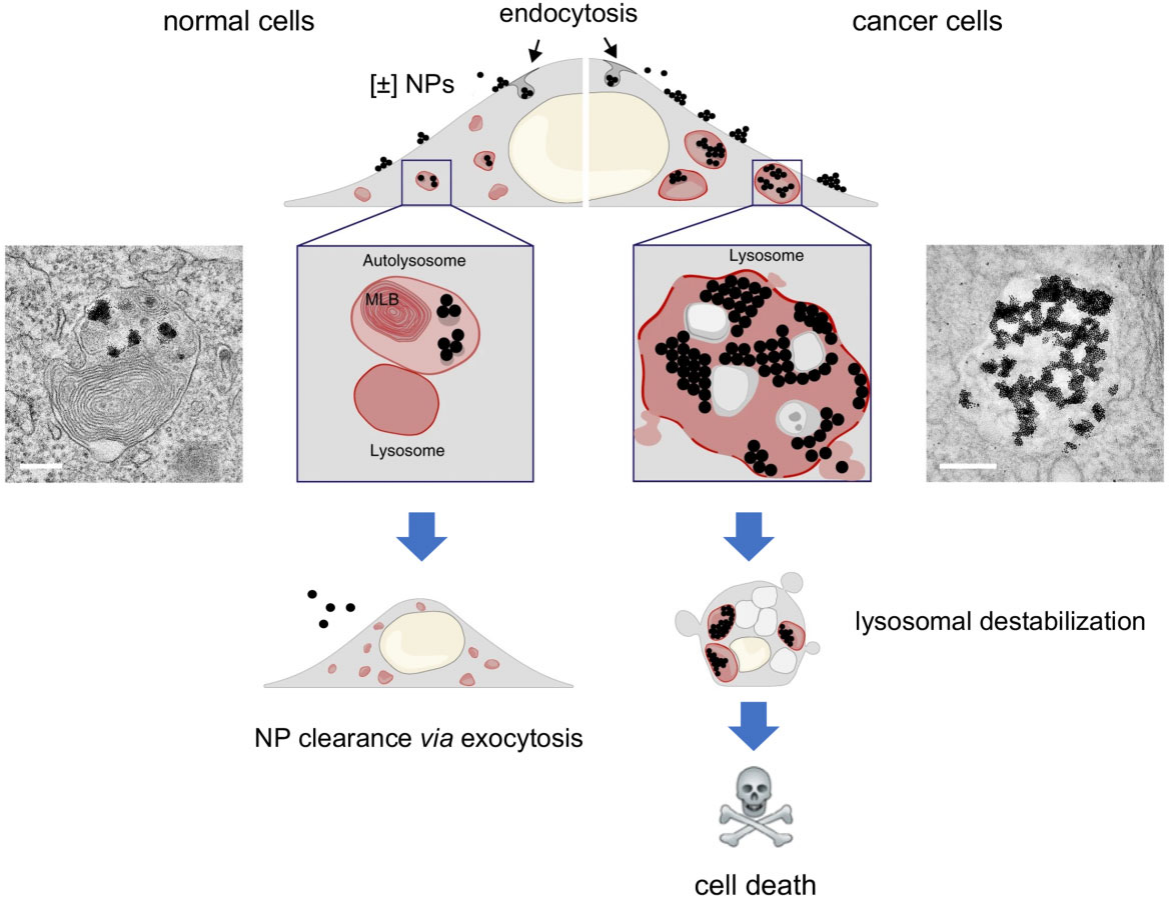
Hijacking the endo-lysosomal system of cancer cells. Borkowska *et al.* (2020) prepared small (5 nm) Au nanoparticles with mixed surface charge by modifying the particle surfaces with positively charged N, N, N-trimethyl(11-mercaptoundecyl)ammonium chloride (TMA) and negatively charged 11-mercaptoundecanoic acid (MUA) at different ratios. These nanoparticles were well dispersed at pH = 7.4 but aggregated to form supraparticles of approximately 2 μm at acidic pH. In addition, once internalized, the nanoparticle aggregates increased the osmotic pressure, forcing lysosomes to swell, ultimately resulting in cell death. In contrast, nanoparticles were expelled by exocytosis in normal (nonmalignant) cells. Thus, by exploiting pH-dependent aggregation of nanoparticles, the authors could selectively kill cancer cells. MLB, multilamellar bodies; NPs, nanoparticles. Scale bars for TEM images: 200 nm. Modified from Borkowska *et al.* (2020) with permission from Springer Nature.

We touched several times on the importance of lysosomes in regulated (necrotic) cell death. However, the popular “suicide bag” hypothesis according to which hydrolytic enzymes are liberated from injured lysosomes resulting in the death of the cell may be overly simplistic (refer to: Clarke, 1990, for a critical discussion). Notwithstanding, the autophagosomal-lysosomal compartment has been implicated in numerous forms of cell death including non-apoptotic cell death, autophagic cell death, and others (Bursch, 2001). Moreover, although mitochondria have been at the center of attention in apoptosis research, recent work has disclosed a previously unrecognized role of lysosomes in the regulation of the canonical mitochondrial apoptosis pathway through the pH-dependent calcium channel RECS1 (Pihán *et al.*, 2021), confirming that the entire cast of organelles are complicit in driving cell death, although mitochondria seem to act as a common conduit for pro-apoptotic stimuli (Ferri and Kroemer, 2001). How is this relevant for nanoparticle-induced cell death? Unlike small molecules, nanoparticles are usually taken up through endocytosis typically leading to substantial accumulation in lysosomes (Mosquera *et al.*, 2018). Nanoparticles can under certain conditions escape from lysosomes, but they may also cause damage to these organelles, leading to cell death (discussed above). Alternatively, nanoparticles in lysosomes might elicit a “lysosomal storage disorder” with impairment of cellular metabolic functions due to the overcrowding of these organelles. Borkowska *et al.* (2020) recently showed that so-called mixed-charge nanoparticles covered with certain ratios of positively and negatively charged ligands were able to selectively target lysosomes in cancer cells while sparing normal cells (Figure 3). The authors could show pH-dependent assembly into nanoparticle aggregates or supracrystals inside lysosomes, followed by lysosomal swelling with gradual loss of integrity of lysosomal membranes and cell death in cancer cells. In contrast, the nanoparticles were extruded from normal cells *via* exocytosis. Modest protection by a caspase-3-selective inhibitor was noted, but cell death was not clarified in detail (Borkowska *et al.*, 2020). Nevertheless, these studies point towards “drug-free” cancer treatment using nanoparticles specifically tailored to exploit the biochemical milieu of cancer cells.

Metal-organic frameworks (MOFs) are porous organic-inorganic hybrid materials that consist of positively charged metal ions surrounded by organic linker molecules. Several groups have deployed MOFs for the induction of ferroptosis and tumor ablation in preclinical models, without gross abnormalities in any major organs, although more detailed toxicity studies are needed (Meng *et al.*, 2019; Zheng *et al.*, 2017). However, the introduction of iron-based nanoparticles into cells does not *a priori* mean that cells will undergo ferroptosis (if this were true then iron-based contrast agents for MRI would hardly make sense). Conversely, ferroptosis could also be triggered by non-iron-based nanoparticles (see, for instance, Zhang *et al.*, 2020a). Alternatively, iron-based nanoparticles could elicit other forms of cell death (and this may depend on the dose). Ploetz *et al.* (2020) demonstrated in a recent study that iron-based MOF nanoparticles were taken up by HeLa cells *via* endocytosis and subsequently caused lysosomal rupture leading to the induction of pyroptosis, as defined by its dependence on caspase-1. However, the term pyroptosis is normally reserved for a form of pro-inflammatory cell death with inflammasome-dependent IL-1β release that occurs in macrophages, typically in response to pathogens, and inflammasome activation was not proven here. In another recent study, He *et al.* (2021) generated optogenetic tools coupled with upconversion nanoparticles to enable wireless (light-induced) control of cancer cell death. The authors were able to trigger light-induced RIPK1 and RIPK3 oligomerization as well as MLKL oligomerization and showed that this could be exploited to trigger tumor cell necroptosis in a xenograft model. Furthermore, they achieved optic control of tumor cell pyroptosis by manipulating GSDMD. These innovative studies set the stage for the precise control of non-apoptotic cell death in cancer cells. Chen *et al.* (2022) synthesized so-called acid-activatable nanophotosensitizers based on amphiphilic block copolymers conjugated with a photosensitizer and a fluorescence quencher to achieve a library of nanoparticles with a distinct pH transition covering the entire pH range of endosome maturation stages. Using this platform, the authors were able to systemically investigate the ability to “tune” the pyroptosis-inducing activity in cancer cells. They could show that the nanophotosensitizers elicited gasdermin E (GSDME)-mediated pyroptosis in GSDME-positive cancer cells. This occurred through oxidative stress in early endosomes leading to the activation of phospholipase C which seemed to drive mitochondria-dependent caspase-3 activation. It is noted that caspase-3-mediated cleavage of GSDME promotes pyroptotic cell death (Wang *et al.*, 2017), thus exemplifying how one form of regulated cell death (apoptosis) can be switched to another, non-apoptotic form of cell death (pyroptosis). These studies pave the way for the rational design of nanoparticles with pyroptosis-triggering activity. However, as pointed out by the authors, the potential long-term toxicity of the nanoparticles needs to be evaluated due to the nondegradable nature of these materials (Chen *et al.*, 2022).

Finally, Xu *et al.* (2022) reported in a very recent study that a copper-based nano-system triggered a non-apoptotic form of cell death in bladder cancer cells denoted as “cuproptosis.” The *in vivo* experimental results indicated that the nano-system caused no gross abnormalities to major organs following i.v. injection while inhibiting tumor growth by more than 90% in a xenograft mouse model of human bladder cancer. Cuproptosis is a novel form of cell death that may be exploited to kill cancer cells due to particular metabolic vulnerabilities (Tsvetkov *et al.*, 2019, 2022).

## Conclusions

Does it matter how cells die? The answer is emphatically, yes. First, if the mode of cell death triggered by a particular nanomaterial can be delineated, we may also be able to devise strategies to circumvent or prevent cell death, thus reducing its toxicity. Second, different cell death modalities differ greatly in terms of their impact on surrounding tissues and on the immune system (Duan *et al.*, 2019; Green *et al.*, 2009). Third, some nanoparticles could potentially be harnessed for therapeutic gain, especially for cancer therapy (Fadeel and Alexiou, 2020), provided that their impact on normal tissues can be kept in check. We hope that we have managed to raise awareness of the different cell death modalities and their implications for nanotoxicological studies with this review. However, there are remaining challenges as we move forward including the fact that the many different varieties of nanomaterials make detailed studies of every material daunting unless high-throughput assays can be designed to take different cell death modalities into account. This is not a trivial matter as it relates to the ways in which we study cell death in (nano)toxicology (in other words, the veracity of the pathways and endpoints that are being studied and the validity or robustness of the assays used to measure them) (Andón and Fadeel, 2013). High-throughput assays for autophagy already exist (Wu *et al.*, 2014), but assays to screen for other forms of regulated cell death are needed to propel the field of nanotoxicology (Fadeel *et al.*, 2018). Finally, although *in vitro* studies have served to shed light on the biological interactions of nanomaterials, the correlation between *in vitro* and *in vivo* outcomes needs to be strengthened (Hussain *et al.*, 2015).

## Declaration of conflicting interests

The authors declared no potential conflicts of interest with respect to the research, authorship, and/or publication of this article.

## References

[kfad008-B1] Andón F. T. , Fadeel B. (2013). Programmed cell death: Molecular mechanisms and implications for safety assessment of nanomaterials. Acc. Chem. Res. 46, 733–742. 22720979 10.1021/ar300020b

[kfad008-B2] Andón F. T. , Mukherjee S. P., Gessner I., Wortmann L., Xiao L., Hultenby K., Shvedova A. A., Mathur S., Fadeel B. (2017). Hollow carbon spheres trigger inflammasome-dependent IL-1β secretion in macrophages. Carbon 113, 243–251.

[kfad008-B3] Balasubramanian K. , Maeda A., Lee J. S., Mohammadyani D., Dar H. H., Jiang J. F., St. Croix C. M., Watkins S., Tyurin V. A., Tyurina Y. Y., et al (2015). Dichotomous roles for externalized cardiolipin in extracellular signaling: Promotion of phagocytosis and attenuation of innate immunity. Sci. Signal. 8, ra95. 26396268 10.1126/scisignal.aaa6179 PMC4760701

[kfad008-B4] Bedoui S. , Herold M. J., Strasser A. (2020). Emerging connectivity of programmed cell death pathways and its physiological implications. Nat. Rev. Mol. Cell Biol. 21, 678–695. 32873928 10.1038/s41580-020-0270-8

[kfad008-B5] Bersuker K. , Hendricks J. M., Li Z., Magtanong L., Ford B., Tang P. H., Roberts M. A., Tong B., Maimone T. J., Zoncu R., et al (2019). The CoQ oxidoreductase FSP1 acts parallel to GPX4 to inhibit ferroptosis. Nature 575, 688–692. 31634900 10.1038/s41586-019-1705-2 PMC6883167

[kfad008-B6] Borges J. P. , Sætra R. S. R., Volchuk A., Bugge M., Devant P., Sporsheim B., Kilburn B. R., Evavold C. L., Kagan J. C., Goldenberg N. M., et al (2022). Glycine inhibits NINJ1 membrane clustering to suppress plasma membrane rupture in cell death. Elife 11, e78609. 36468682 10.7554/eLife.78609 PMC9754625

[kfad008-B7] Borkowska M. , Siek M., Kolygina D. V., Sobolev Y. I., Lach S., Kumar S., Cho Y. K., Kandere-Grzybowska K., Grzybowski B. A. (2020). Targeted crystallization of mixed-charge nanoparticles in lysosomes induces selective death of cancer cells. Nat. Nanotechnol. 15, 331–341. 32203435 10.1038/s41565-020-0643-3

[kfad008-B8] Broz P. , Pelegrín P., Shao F. (2020). The gasdermins, a protein family executing cell death and inflammation. Nat. Rev. Immunol. 20, 143–157. 31690840 10.1038/s41577-019-0228-2

[kfad008-B9] Bursch W. (2001). The autophagosomal-lysosomal compartment in programmed cell death. Cell Death Differ. 8, 569–581. 11536007 10.1038/sj.cdd.4400852

[kfad008-B10] Chen B. , Yan Y., Yang Y., Cao G., Wang X., Wang Y., Wan F., Yin Q., Wang Z., Li Y., et al (2022). A pyroptosis nanotuner for cancer therapy. Nat. Nanotechnol. 17, 788–798. 35606443 10.1038/s41565-022-01125-0

[kfad008-B11] Clarke P. G. (1990). Developmental cell death: Morphological diversity and multiple mechanisms. Anat. Embryol. 181, 195–213. 10.1007/BF00174615 2186664

[kfad008-B12] Danial N. N. , Korsmeyer S. J. (2004). Cell death: Critical control points. Cell 116, 205–219. 14744432 10.1016/s0092-8674(04)00046-7

[kfad008-B13] Degterev A. , Hitomi J., Germscheid M., Ch’en I. L., Korkina O., Teng X., Abbott D., Cuny G. D., Yuan C., Wagner G., et al (2008). Identification of RIP1 kinase as a specific cellular target of necrostatins. Nat. Chem. Biol. 4, 313–321. 18408713 10.1038/nchembio.83 PMC5434866

[kfad008-B14] Degterev A. , Huang Z., Boyce M., Li Y., Jagtap P., Mizushima N., Cuny G. D., Mitchison T. J., Moskowitz M. A., Yuan J. (2005). Chemical inhibitor of nonapoptotic cell death with therapeutic potential for ischemic brain injury. Nat. Chem. Biol. 1, 112–119. 16408008 10.1038/nchembio711

[kfad008-B15] Desai J. , Foresto-Neto O., Honarpisheh M., Steiger S., Nakazawa D., Popper B., Buhl E. M., Boor P., Mulay S. R., Anders H. J. (2017). Particles of different sizes and shapes induce neutrophil necroptosis followed by the release of neutrophil extracellular trap-like chromatin. Sci. Rep. 7, 15003. 29101355 10.1038/s41598-017-15106-0 PMC5670218

[kfad008-B16] Dixon S. J. , Lemberg K. M., Lamprecht M. R., Skouta R., Zaitsev E. M., Gleason C. E., Patel D. N., Bauer A. J., Cantley A. M., Yang W. S., et al (2012). Ferroptosis: An iron-dependent form of nonapoptotic cell death. Cell 149, 1060–1072. 22632970 10.1016/j.cell.2012.03.042 PMC3367386

[kfad008-B17] Doll S. , Freitas F. P., Shah R., Aldrovandi M., da Silva M. C., Ingold I., Goya Grocin A., Xavier da Silva T. N., Panzilius E., Scheel C. H., et al (2019). FSP1 is a glutathione-independent ferroptosis suppressor. Nature 575, 693–698. 31634899 10.1038/s41586-019-1707-0

[kfad008-B18] Drobne D. (2021). Adding toxicological context to nanotoxicity study reporting using the NanoTox metadata list. Small 17, e2005622. 33605049 10.1002/smll.202005622

[kfad008-B19] Du W. , Gu M., Hu M., Pinchi P., Chen W., Ryan M., Nold T., Bannaga A., Xu H. (2021). Lysosomal Zn^2+^ release triggers rapid, mitochondria-mediated, non-apoptotic cell death in metastatic melanoma. Cell Rep. 37, 109848. 34686351 10.1016/j.celrep.2021.109848 PMC8559338

[kfad008-B20] Duan X. , Chan C., Lin W. (2019). Nanoparticle-mediated immunogenic cell death enables and potentiates cancer immunotherapy. Angew. Chem. Int. Ed. Engl. 58, 670–680. 30016571 10.1002/anie.201804882 PMC7837455

[kfad008-B21] Duvall E. , Wyllie A. H. (1986). Death and the cell. Immunol. Today. 7, 115–119. 25289803 10.1016/0167-5699(86)90152-0

[kfad008-B22] Fadeel B. (2019a). The right stuff: On the future of nanotoxicology. Front. Toxicol. 1, 1. 35295768 10.3389/ftox.2019.00001 PMC8915828

[kfad008-B23] Fadeel B. (2019b). Don’t look back in anger: Lessons from cell death research. Biochem. Biophys. Res. Commun. 520, 674–675. 31761070 10.1016/j.bbrc.2019.10.020

[kfad008-B24] Fadeel B. , Alexiou C. (2020). Brave new world revisited: Focus on nanomedicine. Biochem. Biophys. Res. Commun. 533, 36–49. 32921412 10.1016/j.bbrc.2020.08.046

[kfad008-B25] Fadeel B. , Farcal L., Hardy B., Vázquez-Campos S., Hristozov D., Marcomini A., Lynch I., Valsami-Jones E., Alenius H., Savolainen K. (2018). Advanced tools for the safety assessment of nanomaterials. Nat. Nanotechnol. 13, 537–543. 29980781 10.1038/s41565-018-0185-0

[kfad008-B26] Fadeel B. , Orrenius S. (2005). Apoptosis: A basic biological phenomenon with wide-ranging implications in human disease. J. Intern. Med. 258, 479–517. 16313474 10.1111/j.1365-2796.2005.01570.x

[kfad008-B27] Faria M. , Björnmalm M., Thurecht K. J., Kent S. J., Parton R. G., Kavallaris M., Johnston A. P. R., Gooding J. J., Corrie S. R., Boyd B. J., et al (2018). Minimum information reporting in bio-nano experimental literature. Nat. Nanotechnol. 13, 777–785. 30190620 10.1038/s41565-018-0246-4 PMC6150419

[kfad008-B28] Farrera C. , Fadeel B. (2015). It takes two to tango: Understanding the interactions between engineered nanomaterials and the immune system. Eur. J. Pharm. Biopharm. 95, 3–12. 25770769 10.1016/j.ejpb.2015.03.007

[kfad008-B29] Fearnhead H. O. , Vandenabeele P., Vanden Berghe T. (2017). How do we fit ferroptosis in the family of regulated cell death? Cell Death Differ. 24, 1991–1998. 28984871 10.1038/cdd.2017.149 PMC5686356

[kfad008-B30] Ferri K. F. , Kroemer G. (2001). Organelle-specific initiation of cell death pathways. Nat. Cell Biol. 3, E255–E263. 11715037 10.1038/ncb1101-e255

[kfad008-B31] Flores-Romero H. , Ros U., Garcia-Saez A. J. (2020). Pore formation in regulated cell death. EMBO J. 39, e105753. 33124082 10.15252/embj.2020105753 PMC7705454

[kfad008-B32] Gallud A. , Delaval M., Kinaret P., Marwah V. S., Fortino V., Ytterberg J., Zubarev R., Skoog T., Kere J., Correia M., et al (2020). Multiparametric profiling of engineered nanomaterials: Unmasking the surface coating effect. Adv. Sci. 7, 2002221. 10.1002/advs.202002221 PMC7675037 33240770

[kfad008-B33] Gallud A. , Klöditz K., Ytterberg J., Östberg N., Katayama S., Skoog T., Gogvadze V., Chen Y. Z., Xue D., Moya S., et al (2019). Cationic gold nanoparticles elicit mitochondrial dysfunction: A multi-omics study. Sci. Rep. 9, 4366. 30867451 10.1038/s41598-019-40579-6 PMC6416392

[kfad008-B34] Galluzzi L. , Vitale I., Aaronson S. A., Abrams J. M., Adam D., Agostinis P., Alnemri E. S., Altucci L., Amelio I., Andrews D. W., et al (2018). Molecular mechanisms of cell death: Recommendations of the Nomenclature Committee on Cell Death 2018. Cell Death Differ. 25, 486–541. 29362479 10.1038/s41418-017-0012-4 PMC5864239

[kfad008-B35] Green D. R. (2019). The coming decade of cell death research: Five riddles. Cell 177, 1094–1107. 31100266 10.1016/j.cell.2019.04.024 PMC6534278

[kfad008-B36] Green D. R. , Ferguson T., Zitvogel L., Kroemer G. (2009). Immunogenic and tolerogenic cell death. Nat. Rev. Immunol. 9, 353–363. 19365408 10.1038/nri2545 PMC2818721

[kfad008-B37] Green D. R. , Victor B. (2012). The pantheon of the fallen: Why are there so many forms of cell death? Trends Cell Biol. 22, 555–556. 22995729 10.1016/j.tcb.2012.08.008 PMC3568685

[kfad008-B38] Gupta G. , Gliga A., Hedberg J., Serra A., Greco D., Odnevall Wallinder I., Fadeel B. (2020). Cobalt nanoparticles trigger ferroptosis-like cell death (oxytosis) in neuronal cells: Potential implications for neurodegenerative disease. FASEB J. 34, 5262–5281. 32060981 10.1096/fj.201902191RR

[kfad008-B39] Hanahan D. (2022). Hallmarks of cancer: New dimensions. Cancer Discov. 12, 31–46. 35022204 10.1158/2159-8290.CD-21-1059

[kfad008-B40] He B. , Shi Y., Liang Y., Yang A., Fan Z., Yuan L., Zou X., Chang X., Zhang H., Wang X., et al (2018). Single-walled carbon-nanohorns improve biocompatibility over nanotubes by triggering less protein-initiated pyroptosis and apoptosis in macrophages. Nat. Commun. 9, 2393. 29921862 10.1038/s41467-018-04700-z PMC6008334

[kfad008-B41] He L. , Huang Z., Huang K., Chen R., Nguyen N. T., Wang R., Cai X., Huang Z., Siwko S., Walker J. R., et al (2021). Optogenetic control of non-apoptotic cell death. Adv. Sci. 8, 2100424. 10.1002/advs.202100424 PMC8438606 34540558

[kfad008-B42] Hirschhorn T. , Stockwell B. R. (2019). The development of the concept of ferroptosis. Free Radic. Biol. Med. 133, 130–143. 30268886 10.1016/j.freeradbiomed.2018.09.043 PMC6368883

[kfad008-B43] Hitomi J. , Christofferson D. E., Ng A., Yao J., Degterev A., Xavier R. J., Yuan J. (2008). Identification of a molecular signaling network that regulates a cellular necrotic cell death pathway. Cell 135, 1311–1323. 19109899 10.1016/j.cell.2008.10.044 PMC2621059

[kfad008-B44] Holze C. , Michaudel C., Mackowiak C., Haas D. A., Benda C., Hubel P., Pennemann F. L., Schnepf D., Wettmarshausen J., Braun M., et al (2018). Oxeiptosis, a ROS-induced caspase-independent apoptosis-like cell-death pathway. Nat. Immunol. 19, 130–140. 29255269 10.1038/s41590-017-0013-y PMC5786482

[kfad008-B45] Honarpisheh M. , Foresto-Neto O., Desai J., Steiger S., Gómez L. A., Popper B., Boor P., Anders H. J., Mulay S. R. (2017). Phagocytosis of environmental or metabolic crystalline particles induces cytotoxicity by triggering necroptosis across a broad range of particle size and shape. Sci. Rep. 7, 15523. 29138474 10.1038/s41598-017-15804-9 PMC5686194

[kfad008-B46] Hu J. J. , Liu X., Xia S., Zhang Z., Zhang Y., Zhao J., Ruan J., Luo X., Lou X., Bai Y., et al (2020). FDA-approved disulfiram inhibits pyroptosis by blocking gasdermin D pore formation. Nat. Immunol. 21, 736–745. 32367036 10.1038/s41590-020-0669-6 PMC7316630

[kfad008-B47] Huo L. , Chen R., Zhao L., Shi X., Bai R., Long D., Chen F., Zhao Y., Chang Y. Z., Chen C. (2015). Silver nanoparticles activate endoplasmic reticulum stress signaling pathway in cell and mouse models: The role in toxicity evaluation. Biomaterials 61, 307–315. 26024651 10.1016/j.biomaterials.2015.05.029

[kfad008-B48] Husmann M. (2013). Vital dyes and virtual deaths. Cell Death Differ. 20, 963. 23558952 10.1038/cdd.2013.27 PMC3679458

[kfad008-B49] Hussain S. , Sangtian S., Anderson S. M., Snyder R. J., Marshburn J. D., Rice A. B., Bonner J. C., Garantziotis S. (2014). Inflammasome activation in airway epithelial cells after multi-walled carbon nanotube exposure mediates a profibrotic response in lung fibroblasts. Part. Fibre Toxicol. 11, 28. 24915862 10.1186/1743-8977-11-28 PMC4067690

[kfad008-B50] Hussain S. M. , Warheit D. B., Ng S. P., Comfort K. K., Grabinski C. M., Braydich-Stolle L. K. (2015). At the crossroads of nanotoxicology *in vitro*: Past achievements and current challenges. Toxicol. Sci. 147, 5–16. 26310852 10.1093/toxsci/kfv106

[kfad008-B51] Iulianna T. , Kuldeep N., Fossel E. (2022). The Achilles’ heel of cancer: Targeting tumors *via* lysosome-induced immunogenic cell death. Cell Death Dis. 13, 509. 35637197 10.1038/s41419-022-04912-8 PMC9151667

[kfad008-B52] Jeliazkova N. , Apostolova M. D., Andreoli C., Barone F., Barrick A., Battistelli C., Bossa C., Botea-Petcu A., Châtel A., De Angelis I., et al (2021). Towards FAIR nanosafety data. Nat. Nanotechnol. 16, 644–654. 34017099 10.1038/s41565-021-00911-6

[kfad008-B53] Jiang X. , Stockwell B. R., Conrad M. (2021). Ferroptosis: Mechanisms, biology and role in disease. Nat. Rev. Mol. Cell Biol. 22, 266–282. 33495651 10.1038/s41580-020-00324-8 PMC8142022

[kfad008-B54] Kam T. I. , Mao X., Park H., Chou S. C., Karuppagounder S. S., Umanah G. E., Yun S. P., Brahmachari S., Panicker N., Chen R., et al (2018). Poly(ADP-ribose) drives pathologic α-synuclein neurodegeneration in Parkinson’s disease. Science 362, eaat8407. 30385548 10.1126/science.aat8407 PMC6431793

[kfad008-B55] Kambara H. , Liu F., Zhang X., Liu P., Bajrami B., Teng Y., Zhao L., Zhou S., Yu H., Zhou W., et al (2018). Gasdermin D exerts anti-inflammatory effects by promoting neutrophil death. Cell Rep. 22, 2924–2936. 29539421 10.1016/j.celrep.2018.02.067 PMC5878047

[kfad008-B56] Kayagaki N. , Kornfeld O. S., Lee B. L., Stowe I. B., O’Rourke K., Li Q., Sandoval W., Yan D., Kang J., Xu M., et al (2021). NINJ1 mediates plasma membrane rupture during lytic cell death. Nature 591, 131–136. 33472215 10.1038/s41586-021-03218-7

[kfad008-B57] Kayagaki N. , Stowe I. B., Lee B. L., O’Rourke K., Anderson K., Warming S., Cuellar T., Haley B., Roose-Girma M., Phung Q. T., et al (2015). Caspase-11 cleaves gasdermin D for non-canonical inflammasome signalling. Nature 526, 666–671. 26375259 10.1038/nature15541

[kfad008-B58] Keshavan S. , Gupta G., Martin S., Fadeel B. (2021). Multi-walled carbon nanotubes trigger lysosome-dependent cell death (pyroptosis) in macrophages but not in neutrophils. Nanotoxicology 15, 1125–1150. 34657549 10.1080/17435390.2021.1988171

[kfad008-B59] Kim S. E. , Zhang L., Ma K., Riegman M., Chen F., Ingold I., Conrad M., Turker M. Z., Gao M., Jiang X., et al (2016). Ultrasmall nanoparticles induce ferroptosis in nutrient-deprived cancer cells and suppress tumour growth. Nat. Nanotechnol. 11, 977–985. 27668796 10.1038/nnano.2016.164 PMC5108575

[kfad008-B60] Kist M. , Vucic D. (2021). Cell death pathways: Intricate connections and disease implications. EMBO J. 40, e106700. 33439509 10.15252/embj.2020106700 PMC7917554

[kfad008-B61] Klionsky D. J. , Abdel-Aziz A. K., Abdelfatah S., Abdellatif M., Abdoli A., Abel S., Abeliovich H., Abildgaard M. H., Abudu Y. P., Acevedo-Arozena A., et al (2021). Guidelines for the use and interpretation of assays for monitoring autophagy. Autophagy 17, 1–382. 33634751 10.1080/15548627.2020.1797280 PMC7996087

[kfad008-B62] Klöditz K. , Fadeel B. (2019). Three cell deaths and a funeral: Macrophage clearance of cells undergoing distinct modes of cell death. Cell Death Discov. 5, 65. 30774993 10.1038/s41420-019-0146-x PMC6368547

[kfad008-B63] Kluck R. M. , Bossy-Wetzel E., Green D. R., Newmeyer D. D. (1997). The release of cytochrome c from mitochondria: A primary site for Bcl-2 regulation of apoptosis. Science 275, 1132–1136. 9027315 10.1126/science.275.5303.1132

[kfad008-B64] Kono H. , Rock K. L. (2008). How dying cells alert the immune system to danger. Nat. Rev. Immunol. 8, 279–289. 18340345 10.1038/nri2215 PMC2763408

[kfad008-B65] Krammer P. H. (1998). The CD95(APO-1/Fas)/CD95L system. Toxicol. Lett. 102–103, 131–137. 10.1016/s0378-4274(98)00297-5 10022244

[kfad008-B66] Li R. , Ji Z., Chang C. H., Dunphy D. R., Cai X., Meng H., Zhang H., Sun B., Wang X., Dong J., et al (2014a). Surface interactions with compartmentalized cellular phosphates explain rare earth oxide nanoparticle hazard and provide opportunities for safer design. ACS Nano 8, 1771–1783. 24417322 10.1021/nn406166n PMC3988685

[kfad008-B67] Li R. , Ji Z., Qin H., Kang X., Sun B., Wang M., Chang C. H., Wang X., Zhang H., Zou H., et al (2014b). Interference in autophagosome fusion by rare earth nanoparticles disrupts autophagic flux and regulation of an interleukin-1β producing inflammasome. ACS Nano 8, 10280–10292. 25251502 10.1021/nn505002w PMC4213039

[kfad008-B68] Li Y. , Qin T., Ingle T., Yan J., He W., Yin J. J., Chen T. (2017). Differential genotoxicity mechanisms of silver nanoparticles and silver ions. Arch. Toxicol. 91, 509–519. 27180073 10.1007/s00204-016-1730-y

[kfad008-B69] Lison D. , Vietti G., van den Brule S. (2014). Paracelsus in nanotoxicology. Part. Fibre Toxicol. 11, 35. 25138533 10.1186/s12989-014-0035-7 PMC4354280

[kfad008-B70] Liu Y. , Zhu W., Ni D., Zhou Z., Gu J. H., Zhang W., Sun H., Liu F. (2020). Alpha lipoic acid antagonizes cytotoxicity of cobalt nanoparticles by inhibiting ferroptosis-like cell death. J. Nanobiotechnology. 18, 141. 33008409 10.1186/s12951-020-00700-8 PMC7532644

[kfad008-B71] Lu B. , Nakamura T., Inouye K., Li J., Tang Y., Lundbäck P., Valdes-Ferrer S. I., Olofsson P. S., Kalb T., Roth J., et al (2012). Novel role of PKR in inflammasome activation and HMGB1 release. Nature 488, 670–674. 22801494 10.1038/nature11290 PMC4163918

[kfad008-B72] Ma X. , Wu Y., Jin S., Tian Y., Zhang X., Zhao Y., Yu L., Liang X. J. (2011). Gold nanoparticles induce autophagosome accumulation through size-dependent nanoparticle uptake and lysosome impairment. ACS Nano 5, 8629–8639. 21974862 10.1021/nn202155y

[kfad008-B73] MacCormack T. J. , Meli M. V., Ede J. D., Ong K. J., Rourke J. L., Dieni C. A. (2021). Revisiting nanoparticle-assay interference: There’s plenty of room at the bottom for misinterpretation. Comp. Biochem. Physiol. B Biochem. Mol. Biol. 255, 110601. 33857590 10.1016/j.cbpb.2021.110601

[kfad008-B74] Majno G. , Joris I. (1995). Apoptosis, oncosis, and necrosis. An overview of cell death. Am. J. Pathol. 146, 3–15. 7856735 PMC1870771

[kfad008-B75] Martinon F. , Burns K., Tschopp J. (2002). The inflammasome: A molecular platform triggering activation of inflammatory caspases and processing of proIL-β. Mol. Cell 10, 417–426. 12191486 10.1016/s1097-2765(02)00599-3

[kfad008-B76] Meier P. , Vousden K. H. (2007). Lucifer’s labyrinth – ten years of path finding in cell death. Mol. Cell 28, 746–754. 18082600 10.1016/j.molcel.2007.11.016

[kfad008-B77] Meng X. , Deng J., Liu F., Guo T., Liu M., Dai P., Fan A., Wang Z., Zhao Y. (2019). Triggered all-active metal organic framework: Ferroptosis machinery contributes to the apoptotic photodynamic antitumor therapy. Nano Lett. 19, 7866–7876. 31594301 10.1021/acs.nanolett.9b02904

[kfad008-B78] Mirshafiee V. , Sun B., Chang C. H., Liao Y. P., Jiang W., Jiang J., Liu X., Wang X., Xia T., Nel A. E. (2018). Toxicological profiling of metal oxide nanoparticles in liver context reveals pyroptosis in Kupffer cells and macrophages *versus* apoptosis in hepatocytes. ACS Nano 12, 3836–3852. 29543433 10.1021/acsnano.8b01086 PMC5946698

[kfad008-B79] Monteiro-Riviere N. A. , Inman A. O., Zhang L. W. (2009). Limitations and relative utility of screening assays to assess engineered nanoparticle toxicity in a human cell line. Toxicol. Appl. Pharmacol. 234, 222–235. 18983864 10.1016/j.taap.2008.09.030

[kfad008-B80] Mosquera J. , García I., Liz-Marzán L. M. (2018). Cellular uptake of nanoparticles *versus* small molecules: A matter of size. Acc. Chem. Res. 51, 2305–2313. 30156826 10.1021/acs.accounts.8b00292

[kfad008-B81] Mukherjee S. P. , Kostarelos K., Fadeel B. (2018). Cytokine profiling of primary human macrophages exposed to endotoxin-free graphene oxide: Size-independent NLRP3 inflammasome activation. Adv. Healthcare Mater. 7, 1700815. 10.1002/adhm.201700815 29266859

[kfad008-B82] Mulay S. R. , Desai J., Kumar S. V., Eberhard J. N., Thomasova D., Romoli S., Grigorescu M., Kulkarni O. P., Popper B., Vielhauer V., et al (2016). Cytotoxicity of crystals involves RIPK3-MLKL-mediated necroptosis. Nat. Commun. 7, 10274. 26817517 10.1038/ncomms10274 PMC4738349

[kfad008-B83] Munoz L. E. , Bilyy R., Biermann M. H., Kienhöfer D., Maueröder C., Hahn J., Brauner J. M., Weidner D., Chen J., Scharin-Mehlmann M., et al (2016). Nanoparticles size-dependently initiate self-limiting NETosis-driven inflammation. Proc. Natl. Acad. Sci. U. S. A. 113, E5856–E5865. 27647892 10.1073/pnas.1602230113 PMC5056044

[kfad008-B84] Nel A. , Xia T., Mädler L., Li N. (2006). Toxic potential of materials at the nanolevel. Science 311, 622–627. 16456071 10.1126/science.1114397

[kfad008-B85] Newton K. , Dixit V. M., Kayagaki N. (2021). Dying cells fan the flames of inflammation. Science 374, 1076–1080. 34822265 10.1126/science.abi5934

[kfad008-B86] Nicholson D. W. , Ali A., Thornberry N. A., Vaillancourt J. P., Ding C. K., Gallant M., Gareau Y., Griffin P. R., Labelle M., Lazebnik Y. A. (1995). Identification and inhibition of the ICE/CED-3 protease necessary for mammalian apoptosis. Nature 376, 37–43. 7596430 10.1038/376037a0

[kfad008-B87] Ong K. J. , MacCormack T. J., Clark R. J., Ede J. D., Ortega V. A., Felix L. C., Dang M. K., Ma G., Fenniri H., Veinot J. G., et al (2014). Widespread nanoparticle-assay interference: Implications for nanotoxicity testing. PLoS One 9, e90650. 24618833 10.1371/journal.pone.0090650 PMC3949728

[kfad008-B88] Orrenius S. , Nicotera P., Zhivotovsky B. (2011). Cell death mechanisms and their implications in toxicology. Toxicol. Sci. 119, 3–19. 20829425 10.1093/toxsci/kfq268

[kfad008-B89] Overholtzer M. , Mailleux A. A., Mouneimne G., Normand G., Schnitt S. J., King R. W., Cibas E. S., Brugge J. S. (2007). A nonapoptotic cell death process, entosis, that occurs by cell-in-cell invasion. Cell 131, 966–979. 18045538 10.1016/j.cell.2007.10.040

[kfad008-B90] Özkaya A. B. , Geyik C. (2022). From viability to cell death: Claims with insufficient evidence in high-impact cell culture studies. PLoS One 17, e0250754. 35192623 10.1371/journal.pone.0250754 PMC8863264

[kfad008-B91] Palomäki J. , Välimäki E., Sund J., Vippola M., Clausen P. A., Jensen K. A., Savolainen K., Matikainen S., Alenius H. (2011). Long, needle-like carbon nanotubes and asbestos activate the NLRP3 inflammasome through a similar mechanism. ACS Nano 5, 6861–6870. 21800904 10.1021/nn200595c

[kfad008-B92] Peng G. , Keshavan S., Delogu L., Shin Y., Casiraghi C., Fadeel B. (2022). Two-dimensional transition metal dichalcogenides trigger trained immunity in human macrophages through epigenetic and metabolic pathways. Small 18, e2107816. 35434920 10.1002/smll.202107816

[kfad008-B93] Pihán P. , Lisbona F., Borgonovo J., Edwards-Jorquera S., Nunes-Hasler P., Castillo K., Keep O., Urra H., Saarnio S., Vihinen H., et al (2021). Control of lysosomal-mediated cell death by the pH-dependent calcium channel RECS1. Sci. Adv. 7, eabe5469. 34767445 10.1126/sciadv.abe5469 PMC8589314

[kfad008-B94] Ploetz E. , Zimpel A., Cauda V., Bauer D., Lamb D. C., Haisch C., Zahler S., Vollmar A. M., Wuttke S., Engelke H. (2020). Metal-organic framework nanoparticles induce pyroptosis in cells controlled by the extracellular pH. Adv. Mater. 32, e1907267. 32182391 10.1002/adma.201907267

[kfad008-B95] Raff M. C. (1992). Social controls on cell survival and cell death. Nature 356, 397–400. 1557121 10.1038/356397a0

[kfad008-B96] Ranger A. M. , Malynn B. A., Korsmeyer S. J. (2001). Mouse models of cell death. Nat. Genet. 28, 113–118. 11381252 10.1038/88815

[kfad008-B97] Riegman M. , Sagie L., Galed C., Levin T., Steinberg N., Dixon S. J., Wiesner U., Bradbury M. S., Niethammer P., Zaritsky A., et al (2020). Ferroptosis occurs through an osmotic mechanism and propagates independently of cell rupture. Nat. Cell Biol. 22, 1042–1048. 32868903 10.1038/s41556-020-0565-1 PMC7644276

[kfad008-B98] Rizzollo F. , More S., Vangheluwe P., Agostinis P. (2021). The lysosome as a master regulator of iron metabolism. Trends Biochem. Sci. 46, 960–975. 34384657 10.1016/j.tibs.2021.07.003

[kfad008-B99] Rohde M. M. , Snyder C. M., Sloop J., Solst S. R., Donati G. L., Spitz D. R., Furdui C. M., Singh R. (2021). The mechanism of cell death induced by silver nanoparticles is distinct from silver cations. Part. Fibre Toxicol. 18, 37. 34649580 10.1186/s12989-021-00430-1 PMC8515661

[kfad008-B100] Rühl S. , Shkarina K., Demarco B., Heilig R., Santos J. C., Broz P. (2018). ESCRT-dependent membrane repair negatively regulates pyroptosis downstream of GSDMD activation. Science 362, 956–960. 30467171 10.1126/science.aar7607

[kfad008-B101] Sagawa T. , Honda A., Ishikawa R., Miyasaka N., Nagao M., Akaji S., Kida T., Tsujikawa T., Yoshida T., Kawahito Y., et al (2021). Role of necroptosis of alveolar macrophages in acute lung inflammation of mice exposed to titanium dioxide nanoparticles. Nanotoxicology 15, 1312–1330. 35000540 10.1080/17435390.2021.2022231

[kfad008-B102] Seiler A. , Schneider M., Förster H., Roth S., Wirth E. K., Culmsee C., Plesnila N., Kremmer E., Rådmark O., Wurst W., et al (2008). Glutathione peroxidase 4 senses and translates oxidative stress into 12/15-lipoxygenase dependent- and AIF-mediated cell death. Cell Metab. 8, 237–248. 18762024 10.1016/j.cmet.2008.07.005

[kfad008-B103] Shi J. , Zhao Y., Wang K., Shi X., Wang Y., Huang H., Zhuang Y., Cai T., Wang F., Shao F. (2015). Cleavage of GSDMD by inflammatory caspases determines pyroptotic cell death. Nature 526, 660–665. 26375003 10.1038/nature15514

[kfad008-B104] Soriano-Castell D. , Currais A., Maher P. (2021). Defining a pharmacological inhibitor fingerprint for oxytosis/ferroptosis. Free Radic. Biol. Med. 171, 219–231. 34010663 10.1016/j.freeradbiomed.2021.05.023 PMC8217321

[kfad008-B105] Stockwell B. R. (2022). Ferroptosis turns 10: Emerging mechanisms, physiological functions, and therapeutic applications. Cell 185, 2401–2421. 35803244 10.1016/j.cell.2022.06.003 PMC9273022

[kfad008-B106] Stockwell B. R. , Friedmann Angeli J. P., Bayir H., Bush A. I., Conrad M., Dixon S. J., Fulda S., Gascón S., Hatzios S. K., Kagan V. E., et al (2017). Ferroptosis: A regulated cell death nexus linking metabolism, redox biology, and disease. Cell 171, 273–285. 28985560 10.1016/j.cell.2017.09.021 PMC5685180

[kfad008-B107] Stoyanovsky D. A. , Tyurina Y. Y., Shrivastava I., Bahar I., Tyurin V. A., Protchenko O., Jadhav S., Bolevich S. B., Kozlov A. V., Vladimirov Y. A., et al (2019). Iron catalysis of lipid peroxidation in ferroptosis: Regulated enzymatic or random free radical reaction? Free Radic. Biol. Med. 133, 153–161. 30217775 10.1016/j.freeradbiomed.2018.09.008 PMC6555767

[kfad008-B108] Sun B. , Wang X., Ji Z., Wang M., Liao Y. P., Chang C. H., Li R., Zhang H., Nel A. E., Xia T. (2015). NADPH oxidase-dependent NLRP3 inflammasome activation and its important role in lung fibrosis by multiwalled carbon nanotubes. Small 11, 2087–2097. 25581126 10.1002/smll.201402859 PMC4420651

[kfad008-B109] Sun L. , Wang H., Wang Z., He S., Chen S., Liao D., Wang L., Yan J., Liu W., Lei X., et al (2012). Mixed lineage kinase domain-like protein mediates necrosis signaling downstream of RIP3 kinase. Cell 148, 213–227. 22265413 10.1016/j.cell.2011.11.031

[kfad008-B110] Swanson K. V. , Deng M., Ting J. P. (2019). The NLRP3 inflammasome: Molecular activation and regulation to therapeutics. Nat. Rev. Immunol. 19, 477–489. 31036962 10.1038/s41577-019-0165-0 PMC7807242

[kfad008-B111] Szwed M. , Sønstevold T., Øverbye A., Engedal N., Grallert B., Mørch Y., Sulheim E., Iversen T. G., Skotland T., Sandvig K., et al (2019). Small variations in nanoparticle structure dictate differential cellular stress responses and mode of cell death. Nanotoxicology 13, 761–782. 30760074 10.1080/17435390.2019.1576238

[kfad008-B112] Takahashi N. , Duprez L., Grootjans S., Cauwels A., Nerinckx W., DuHadaway J. B., Goossens V., Roelandt R., Van Hauwermeiren F., Libert C., et al (2012). Necrostatin-1 analogues: Critical issues on the specificity, activity and *in vivo* use in experimental disease models. Cell Death Dis. 3, e437. 23190609 10.1038/cddis.2012.176 PMC3542611

[kfad008-B113] Tewari M. , Quan L. T., O’Rourke K., Desnoyers S., Zeng Z., Beidler D. R., Poirier G. G., Salvesen G. S., Dixit V. M. (1995). Yama/CPP32 beta, a mammalian homolog of CED-3, is a CrmA-inhibitable protease that cleaves the death substrate poly(ADP-ribose) polymerase. Cell 81, 801–809. 7774019 10.1016/0092-8674(95)90541-3

[kfad008-B114] Thapa R. J. , Nogusa S., Chen P., Maki J. L., Lerro A., Andrake M., Rall G. F., Degterev A., Balachandran S. (2013). Interferon-induced RIP1/RIP3-mediated necrosis requires PKR and is licensed by FADD and caspases. Proc. Natl. Acad. Sci. U. S. A. 110, E3109–E3118. 23898178 10.1073/pnas.1301218110 PMC3746924

[kfad008-B115] Torii S. , Shintoku R., Kubota C., Yaegashi M., Torii R., Sasaki M., Suzuki T., Mori M., Yoshimoto Y., Takeuchi T., et al (2016). An essential role for functional lysosomes in ferroptosis of cancer cells. Biochem. J. 473, 769–777. 26759376 10.1042/BJ20150658

[kfad008-B116] Trump B. F. , Berezesky I. K., Chang S. H., Phelps P. C. (1997). The pathways of cell death: Oncosis, apoptosis, and necrosis. Toxicol. Pathol. 25, 82–88. 9061857 10.1177/019262339702500116

[kfad008-B117] Tsai Y. Y. , Huang Y. H., Chao Y. L., Hu K. Y., Chin L. T., Chou S. H., Hour A. L., Yao Y. D., Tu C. S., Liang Y. J., et al (2011). Identification of the nanogold particle-induced endoplasmic reticulum stress by omic techniques and systems biology analysis. ACS Nano 5, 9354–9369. 22107733 10.1021/nn2027775

[kfad008-B118] Tsvetkov P. , Coy S., Petrova B., Dreishpoon M., Verma A., Abdusamad M., Rossen J., Joesch-Cohen L., Humeidi R., Spangler R. D., et al (2022). Copper induces cell death by targeting lipoylated TCA cycle proteins. Science 375, 1254–1261. 35298263 10.1126/science.abf0529 PMC9273333

[kfad008-B119] Tsvetkov P. , Detappe A., Cai K., Keys H. R., Brune Z., Ying W., Thiru P., Reidy M., Kugener G., Rossen J., et al (2019). Mitochondrial metabolism promotes adaptation to proteotoxic stress. Nat. Chem. Biol. 15, 681–689. 31133756 10.1038/s41589-019-0291-9 PMC8183600

[kfad008-B120] Turk B. , Turk V. (2009). Lysosomes as “suicide bags” in cell death: Myth or reality? J. Biol. Chem. 284, 21783–21787. 19473965 10.1074/jbc.R109.023820 PMC2755904

[kfad008-B121] Vanden Berghe T. , Linkermann A., Jouan-Lanhouet S., Walczak H., Vandenabeele P. (2014). Regulated necrosis: The expanding network of non-apoptotic cell death pathways. Nat. Rev. Mol. Cell Biol. 15, 135–147. 24452471 10.1038/nrm3737

[kfad008-B122] Vercammen D. , Beyaert R., Denecker G., Goossens V., Van Loo G., Declercq W., Grooten J., Fiers W., Vandenabeele P. (1998). Inhibition of caspases increases the sensitivity of L929 cells to necrosis mediated by tumor necrosis factor. J. Exp. Med. 187, 1477–1485. 9565639 10.1084/jem.187.9.1477 PMC2212268

[kfad008-B123] Wang J. , Chen H. J., Hang T., Yu Y., Liu G., He G., Xiao S., Yang B. R., Yang C., Liu F., et al (2018). Physical activation of innate immunity by spiky particles. Nat. Nanotechnol. 13, 1078–1086. 30374159 10.1038/s41565-018-0274-0 PMC7432992

[kfad008-B124] Wang X. , Chang C. H., Jiang J., Liu X., Li J., Liu Q., Liao Y. P., Li L., Nel A. E., Xia T. (2020). Mechanistic differences in cell death responses to metal-based engineered nanomaterials in Kupffer cells and hepatocytes. Small 16, e2000528. 32337854 10.1002/smll.202000528 PMC7263057

[kfad008-B125] Wang Y. , An R., Umanah G. K., Park H., Nambiar K., Eacker S. M., Kim B., Bao L., Harraz M. M., Chang C., et al (2016). A nuclease that mediates cell death induced by DNA damage and poly(ADP-ribose) polymerase-1. Science 354, aad6872. 27846469 10.1126/science.aad6872 PMC5134926

[kfad008-B126] Wang Y. , Gao W., Shi X., Ding J., Liu W., He H., Wang K., Shao F. (2017). Chemotherapy drugs induce pyroptosis through caspase-3 cleavage of a gasdermin. Nature 547, 99–103. 28459430 10.1038/nature22393

[kfad008-B127] Watson C. Y. , DeLoid G. M., Pal A., Demokritou P. (2016). Buoyant nanoparticles: Implications for nano-biointeractions in cellular studies. Small 12, 3172–3180. 27135209 10.1002/smll.201600314 PMC5089376

[kfad008-B128] Weber R. A. , Yen F. S., Nicholson S. P. V., Alwaseem H., Bayraktar E. C., Alam M., Timson R. C., La K., Abu-Remaileh M., Molina H., et al (2020). Maintaining iron homeostasis is the key role of lysosomal acidity for cell proliferation. Mol. Cell 77, 645–655.e7. 31983508 10.1016/j.molcel.2020.01.003 PMC7176020

[kfad008-B129] Whyte M. , Evan G. (1995). Apoptosis. The last cut is the deepest. Nature 376, 17–18. 7596422 10.1038/376017a0

[kfad008-B130] Wiernicki B. , Dubois H., Tyurina Y. Y., Hassannia B., Bayir H., Kagan V. E., Vandenabeele P., Wullaert A., Vanden Berghe T. (2020). Excessive phospholipid peroxidation distinguishes ferroptosis from other cell death modes including pyroptosis. Cell Death Dis. 11, 922. 33110056 10.1038/s41419-020-03118-0 PMC7591475

[kfad008-B131] Wiernicki B. , Maschalidi S., Pinney J., Adjemian S., Vanden Berghe T., Ravichandran K. S., Vandenabeele P. (2022). Cancer cells dying from ferroptosis impede dendritic cell-mediated anti-tumor immunity. Nat. Commun. 13, 3676. 35760796 10.1038/s41467-022-31218-2 PMC9237053

[kfad008-B132] Wu L. , Zhang Y., Zhang C., Cui X., Zhai S., Liu Y., Li C., Zhu H., Qu G., Jiang G., et al (2014). Tuning cell autophagy by diversifying carbon nanotube surface chemistry. ACS Nano 8, 2087–2099. 24552177 10.1021/nn500376w PMC5586106

[kfad008-B133] Wyllie A. H. (1980). Glucocorticoid-induced thymocyte apoptosis is associated with endogenous endonuclease activation. Nature 284, 555–556. 6245367 10.1038/284555a0

[kfad008-B134] Xu S. , Zheng H., Ma R., Wu D., Pan Y., Yin C., Gao M., Wang W., Li W., Liu S., et al (2020). Vacancies on 2D transition metal dichalcogenides elicit ferroptotic cell death. Nat. Commun. 11, 3484. 32661253 10.1038/s41467-020-17300-7 PMC7359333

[kfad008-B135] Xu Y. , Liu S. Y., Zeng L., Ma H., Zhang Y., Yang H., Liu Y., Fang S., Zhao J., Xu Y., et al (2022). An enzyme-engineered nonporous copper(I) coordination polymer nanoplatform for cuproptosis-based synergistic cancer therapy. Adv. Mater. 34, e2204733. 36054475 10.1002/adma.202204733

[kfad008-B136] Yang J. , Liu X., Bhalla K., Kim C. N., Ibrado A. M., Cai J., Peng T. I., Jones D. P., Wang X. (1997). Prevention of apoptosis by Bcl-2: Release of cytochrome c from mitochondria blocked. Science 275, 1129–1132. 9027314 10.1126/science.275.5303.1129

[kfad008-B137] Yang W. S. , Ramaratnam R., Welsch M. E., Shimada K., Skouta R., Viswanathan V. S., Cheah J. H., Clemons P. A., Shamji A. F., Clish C. B., et al (2014). Regulation of ferroptotic cancer cell death by GPX4. Cell 156, 317–331. 24439385 10.1016/j.cell.2013.12.010 PMC4076414

[kfad008-B138] Yang W. S. , Stockwell B. R. (2008). Synthetic lethal screening identifies compounds activating iron-dependent, nonapoptotic cell death in oncogenic-RAS-harboring cancer cells. Chem. Biol. 15, 234–245. 18355723 10.1016/j.chembiol.2008.02.010 PMC2683762

[kfad008-B139] Zhang C. , Liu Z., Zhang Y., Ma L., Song E., Song Y. (2020a). “Iron free” zinc oxide nanoparticles with ion-leaking properties disrupt intracellular ROS and iron homeostasis to induce ferroptosis. Cell Death Dis. 11, 183. 32170066 10.1038/s41419-020-2384-5 PMC7070056

[kfad008-B140] Zhang F. , Hou Y., Zhu M., Deng B., Zhao M., Zhu X., Sun Y., Chen D., Jiang C., Wang L., et al (2021). Death pathways of cancer cells modulated by surface molecule density on gold nanorods. Adv. Sci. 8, e2102666. 10.1002/advs.202102666 PMC8596106 34523247

[kfad008-B141] Zhang M. , Song R., Liu Y., Yi Z., Meng X., Zhang J., Tang Z., Yao Z., Liu Y., Liu X., et al (2019). Calcium-overload-mediated tumor therapy by calcium peroxide nanoparticles. Chem 5, 2171–2182.

[kfad008-B142] Zhang R. , Piao M. J., Kim K. C., Kim A. D., Choi J. Y., Choi J., Hyun J. W. (2012). Endoplasmic reticulum stress signaling is involved in silver nanoparticles-induced apoptosis. Int. J. Biochem. Cell Biol. 44, 224–232. 22064246 10.1016/j.biocel.2011.10.019

[kfad008-B143] Zhang T. , Yin C., Boyd D. F., Quarato G., Ingram J. P., Shubina M., Ragan K. B., Ishizuka T., Crawford J. C., Tummers B., et al (2020b). Influenza virus Z-RNAs induce ZBP1-mediated necroptosis. Cell 180, 1115–1129.e13. 32200799 10.1016/j.cell.2020.02.050 PMC7153753

[kfad008-B144] Zhao J. , Jitkaew S., Cai Z., Choksi S., Li Q., Luo J., Liu Z. G. (2012). Mixed lineage kinase domain-like is a key receptor interacting protein 3 downstream component of TNF-induced necrosis. Proc. Natl. Acad. Sci. U.S.A. 109, 5322–5327. 22421439 10.1073/pnas.1200012109 PMC3325682

[kfad008-B145] Zheng D. W. , Lei Q., Zhu J. Y., Fan J. X., Li C. X., Li C., Xu Z., Cheng S. X., Zhang X. Z. (2017). Switching apoptosis to ferroptosis: Metal-organic network for high-efficiency anticancer therapy. Nano Lett. 17, 284–291. 28027643 10.1021/acs.nanolett.6b04060

[kfad008-B146] Zilka O. , Shah R., Li B., Friedmann Angeli J. P., Griesser M., Conrad M., Pratt D. A. (2017). On the mechanism of cytoprotection by ferrostatin-1 and liproxstatin-1 and the role of lipid peroxidation in ferroptotic cell death. ACS Cent. Sci. 3, 232–243. 28386601 10.1021/acscentsci.7b00028 PMC5364454

